# ‘Roly-poly toy’ motion during pollen exudation promotes rapid pollen adhesion in rice

**DOI:** 10.1038/s42003-025-08018-7

**Published:** 2025-04-18

**Authors:** Hiroshi Wada, Yuto Hatakeyama, Rosa Erra-Balsells, Takumi Muneta, Hiroshi Nonami, Hikari Ueda, Yoko Yamaga-Hatakeyama, Naoya Miyashita, Takuya Araki

**Affiliations:** 1 Graduate School of Agriculture, Ehime University, Matsuyama, Ehime Japan; 2 Kyushu Okinawa Agricultural Research Center, National Agriculture and Food Research Organization, Chikugo, Fukuoka Japan; 3 Department of Organic Chemistry and CIHIDECAR-CONICET, University of Buenos Aires, Buenos Aires, Argentina; 4 Sumika Agrotech Corporation Ltd, Oyama, Tochigi Japan; 5 The United Graduate School of Agricultural Sciences, Ehime University, Matsuyama, Ehime Japan

**Keywords:** Pollination, Plant physiology, Plant cell biology

## Abstract

In angiosperm, successful pollen adhesion and hydration on the stigma are essential for pollen germination and tube elongation. Self-pollinated grass plants, such as rice, exhibit viscous ‘pollen exudation’ prior to adhesion; however, its cellular dynamics, including their chemical composition, remain unknown. Here, we revisit pollen exudation in rice to find that pollen grains showed ‘Roly-poly toy’-like rocking motion on the exudates to lead pollen adhesion. Single-cell metabolomics revealed that exudates were composed of high content of sugar together with fatty acids and redox-related metabolites, different from mature pollen grains and stigma cells. And hence, these solutes might participate in osmotic and molecular signaling in stigmatic apoplast, increasing the fluid viscosity. Taken together, it is concluded that the unique behaviour observed in rice pollen grains might play a crucial role on optimal self-positioning and adhesion prior to pollen germination, resulting in the rapid self-pollination.

## Abstract (web summary)

A study on rice pollination using single-cell metabolomics and microscopy reports a discovery such that pollen grains attached show “Roly-poly toy”-like physical motion subsequently after exudation, leading to pollen adhesion and germination to facilitate rapid pollination.

## Introduction

**Fig. 1:**
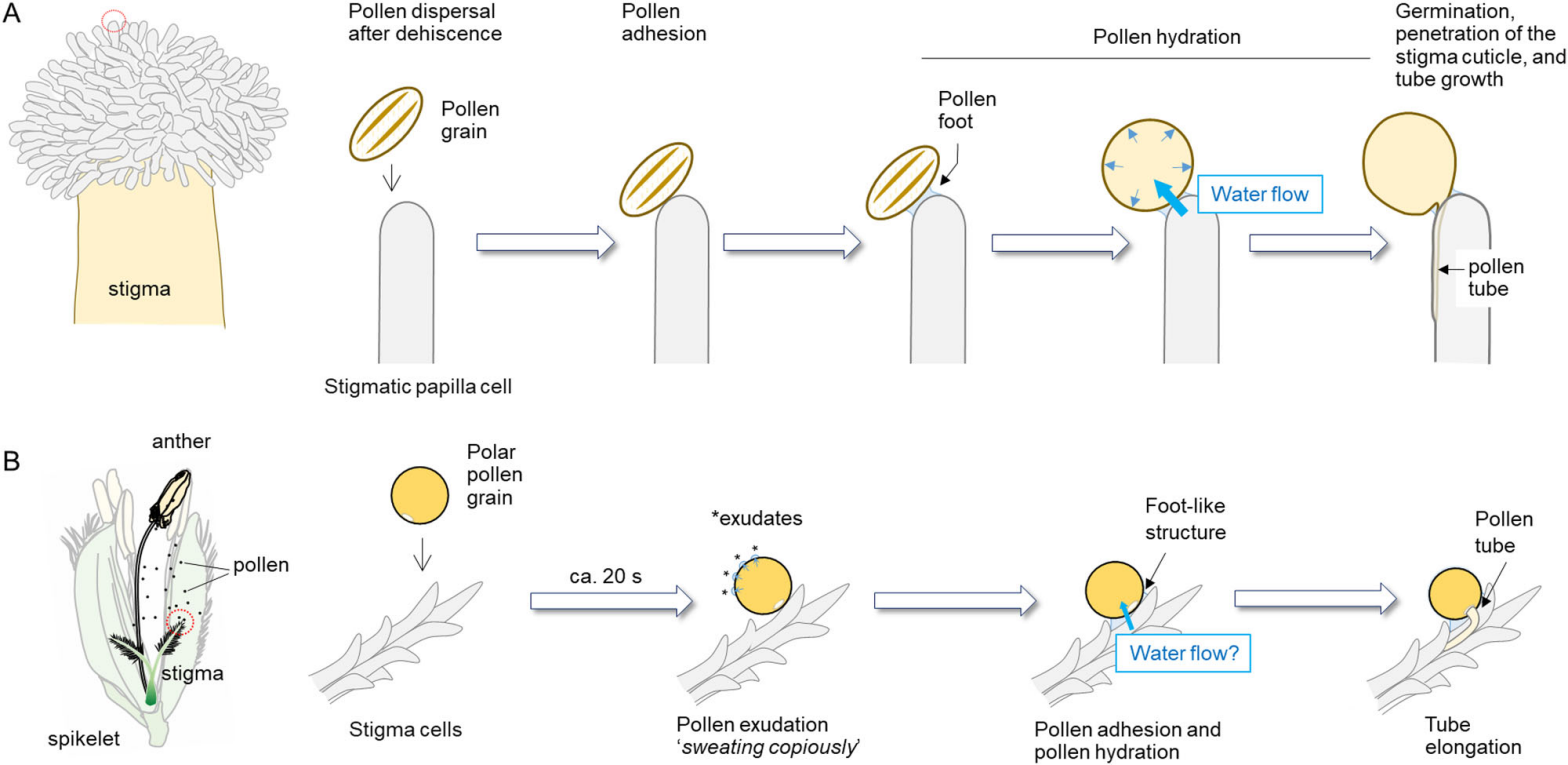
Contrasting differences in pollen-stigma interaction during pollination in *Arabidopsis thaliana* and *Oryza sativa.* Each cartoon for pollination process in *A. thaliana* (**A**) and *O. sativa* (**B**) was drawn based on Chapman and Goring (2010) and Watanabe (1955), respectively, with a partial modification.

In plantae, the success of angiosperms in achieving dominant species over the wide range of global surface is mainly due to the evolutional development of reproduction and vascular systems under upland conditions. Angiosperms reproduce throughout a process called pollination, at which pollen grains move from anthers to stigma, for reproduction to take place. In most flowering plants, pollen grains are partially or fully dehydrated and not metabolically active at pollen dispersal. After pollen grains reach to stigma papillae in *Arabidopsis thaliana* (i.e. pollen capture), it has been accepted that pollen adhesion occurs at the interface between pollen and stigma, followed by pollen hydration according to the water potential gradient throughout the newly formed ‘pollen foot’ that is originated from pollen coat^1,2^ (Fig. 1A). And subsequently, a dramatic increase in pollen grain volume (*V*_*pollen*_) occurs due to the water supply from the stigma and signalling cascade is induced, resulting in germination, stigmatic penetration, and tube growth^1,2^ (Fig. 1A).

Gramineae (grass family) typically exhibits hydrating pollen grains, which sustains a high level of metabolic activity at pollen release from dehiscent anther^3–5^. In contrast to the pollination pattern in *A. thaliana* (Fig. 1A), few papers reported that grass pollen grains *sweat* on the stigma attached shortly after pollen capture^6–9^ (Fig. 1B). This liquid exudation, observed as the earliest event of grass pollination, was termed ‘pollen exudation’^6^. It has been suggested that the exudates originated from the germ pore and non-apertural exine might be attributed to a rapid increase in permeability in the grains^6^, implying rapid changes in pollen metabolism upon pollen capture. It was also suggested that the extent of exudation might be closely associated with the ability of germination^7^. Despite the physiological importance, pollen exudation in grass plants has long been overlooked, presumably due to the rapidness and fineness of event^1,8–11^. And thus, the exact roles, including the chemical composition, remain unclear.

Currently, extremely high temperature conditions at flowering have been frequently disrupting spikelet fertility in grass production areas to cause yield instability^12–14^. In rice, the exposure to high temperatures (≥35 °C) at flowering often causes spikelet sterility in the growth chamber experiment^15^. A lack of pollen viability^4,16^, insufficient thecae dehiscence due to inadequate pollen swelling^17^, a reduction in the number of pollen grains on stigma^12^ have been pointed as the major causes of heat-induced spikelet sterility. Of these, the number of pollen grains on the stigma has been evaluated, although what causes the reduction remains questionable. Considering the sequence of each event in grass pollination described above (Fig. 1B), it is possible that pollen exudation might be the prerequisite of pollen adhesion on the stigma. Revealing the exact pollen behaviour as well as the chemical composition in picolitre exudates may provide useful information to address the heat-related damage at flowering.

During the last decade, single-cell metabolomics for such small liquid samples has been advanced by the improvement of various soft ionisation techniques and the addition to the mass analysers, such as Orbitrap mass spectrometer^18^. Of these, ‘picolitre pressure-probe electrospray-ionisation mass spectrometry’ (picoPPESI-MS) has been developed by combining a cell water status apparatus with a built-in pressure sensor, called *cell pressure probe* (CPP), with an Orbitrap mass spectrometer as mass analyser^19,20^(see Introduction in ref. ^19^). The CPP oil-filled microcapillary allows to sample selected volumes of cell sap, exudates, etc. In order to conduct the electrospray (ESI) volatilisation/ionisation process, an internal electrode has been embedded into the CPP capillary holder in picoPPESI-MS. Thus, an oil-filled microcapillary tip containing the needle electrode is constructed. High-voltage can be directly applied to the collected fluids for ESI volatilisation/ionisation and MS analysis^19^. PicoPPESI-MS allows to analyse the chemical composition in picolitre crude cellular fluids directly collected from the target cells in growing plants, as shown previously^4,19–25^. In this work, we revisit the process of pollen exudation, conducting microscopic observations and picoPPESI-MS analysis in the picolitre fluids. Here we present the details of the unique pollen behaviour occurring at the early step of rice pollination, together with the chemical composition in exudates. The possible intracellular causes regarding the observed pollen behaviour will also be discussed.

## Results

### Pollen behaviour during rice pollination

**Fig. 2:**
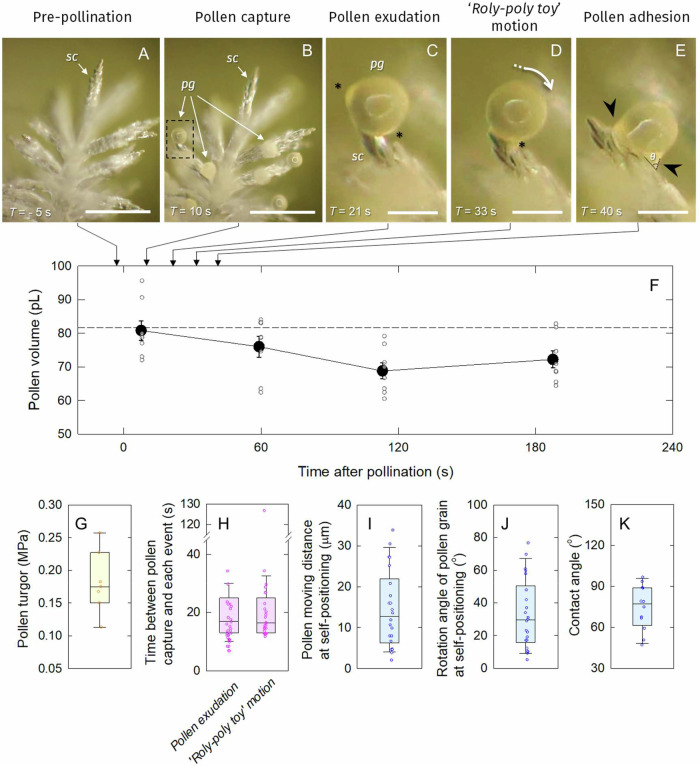
Dynamics of pollen exudation and *roly-poly toy*-like motion during the early post-pollination in rice. Microscopic observations at each event, pre-pollination (**A**), pollen capture (**B**), pollen exudation (**C**), *roly-poly toy*-like motion (**D**) and pollen adhesion (**E**) photographed during artificial pollination in rice (cv. ‘Koshihikari’). In (**F**) time-course of changes in *V*_*pollen*_ on the stigma after pollination. **C**, **E** are the expanded images taken at a part of stigma shown with the dashed rectangle in (**B**). *sc* and *pg* indicate stigma cells and a pollen grain, respectively. Shortly after pollen capture, exudation occurs from pollen surface (see asterisks in **C**, **D**), followed by self-positioning (shown by white arrow) to be tightly fixed at the certain position on stigma by adhesive surface tension effect of the liquid, forming pollen foot (see black arrowheads in **E**), leading to germination. The contact angle, *θ* was shown in (**E**). Data in F is mean ± SE for seven pollen grains from 3 independent plants, and the putative average *V*_*pollen*_ at pollen capture is shown in dashed line in (**F**). Box-plots for pollen turgor prior to anther dehiscence (**G**), times from pollen capture to the initiation of pollen exudation or *roly-poly toy* motion (**H**), pollen moving distance (**I**) and rotation angle (**J**) at self-positioning, and the contact angle calculated at pollen adhesion (**K**). Bars in **A**, **B**, **C**–**E** show 200 μm and 40 μm, respectively. Components of boxplots in **G**–**K** are: center line, median; box limits, upper and lower quartiles; whiskers, 1.5x interquartile range; error range: highest and lowest values excluding outliers.

With the dehiscing anthers in intact plants, artificial pollination was conducted on the stigma in the attached spikelets, and subsequently a close inspection was made (Fig. 2A–E, also see Supplementary Movie 1). In contrast to *Arabidopsis thaliana* (Fig. 1A), rice pollination showed a completely different pattern after landed on stigma (Figs. 1B and 2). Having a lapse of *apparent* stationary state after pollen capture, multiple aquatic picolitre exudates appeared at the grain surface at approximately 20 s after pollen capture, and these *sweat*-like exudates are repeatedly secreted (Fig. 2C and H, and Supplementary Movie 1). These exudates merged to run down (overlaid) throughout the empty space of the exine towards the contact site (Fig. 2C), as the transparency in pollen grains instantly increased (see 00’22” in Supplementary Movie 1). And subsequently, each pollen grain was shown to rock either back and/or forth with a winding path (see Fig. S3C, D, and Supplementary Movie 2), sliding on the stigma surface behaving like a *roly-poly toy* (Fig. 2D) on the lubricant exudates with subsequent small exudation, which resulted in the optimal self-positioning and adhesion of pollen grains to the stigma covered with exudates (Fig. 2D, E). In cv. ‘Koshihikari’, *V*_*pollen*_ at pollen capture was 80.8 pL (Fig. 2F), and during the first 2 min after pollination *V*_*pollen*_ continuously decreased down to 85.3% of initial *V*_*pollen*_ (Fig. 2F). And thereafter, *V*_*pollen*_ slightly increased, reaching 89.5% of initial *V*_*pollen*_ (Fig. 2F).

The number of mature pollen grains per anther and pollen grains adhered on stigma in Koshihikari used was 1248 and 142 on average, respectively. Pollen grain turgor prior to anther dehiscence was 0.18 ± 0.05 MPa (*n* = 7, ranging between 0.15 and 0.23 MPa) (Fig. 2G). Spikelet fertility in Koshihikari used in this study was 96.0% on average (*n* = 7). The time required for the initiation of pollen exudation and the rocking motion after pollen capture was 19 s (*n* = 29, ranging between 8 and 44 s) and 23 s (*n* = 26, ranging between 12 and 126 s), respectively (Fig. 2H). In addition, the image analysis shows that the moving distance of grains observed before and after the rocking motion (self-positioning) was 14.5 ± 9.4 μm (Fig. 2I). The rotation angle during the motion was 32.7° ± 21.3° (*n* = 22, ranging between 5° and 77°, Fig. 2J). The average contact angle formed between the exudate and stigma surface at pollen adhesion (see *θ* in Fig. 2E) was 74.4 ± 16.4° (*n* = 12, ranging between 47.1° and 96.7°) (Fig. 2K).

In addition, in-oil and on-tape microscopic observations were individually conducted to test if the presence of stigma was required for pollen exudation in intact rice plants. In the in-oil experiment, pollen exudation initiated between 20 and 25 s after dipped into the oil, and the exudates were repeatedly secreted (Fig. S1A, B) to be merged to cover the entire grains (Fig. S1C, D), similar to the pollen grains on stigma (Fig. 2C–E). And thereafter, pollen germination occurred at ~120 s after dipped into the oil (Fig. S1D, E). When the grains were placed on the adhesive tape under the ambient conditions (Fig. S1F), pollen exudation similarly occurred from the grain surface. Exudation was observed to last for about 90 s, and thereafter pollen grains gradually shrank due to dehydration (Fig. S1F–J).

### Site-specific differences in metabolites at pollination

**Fig. 3:**
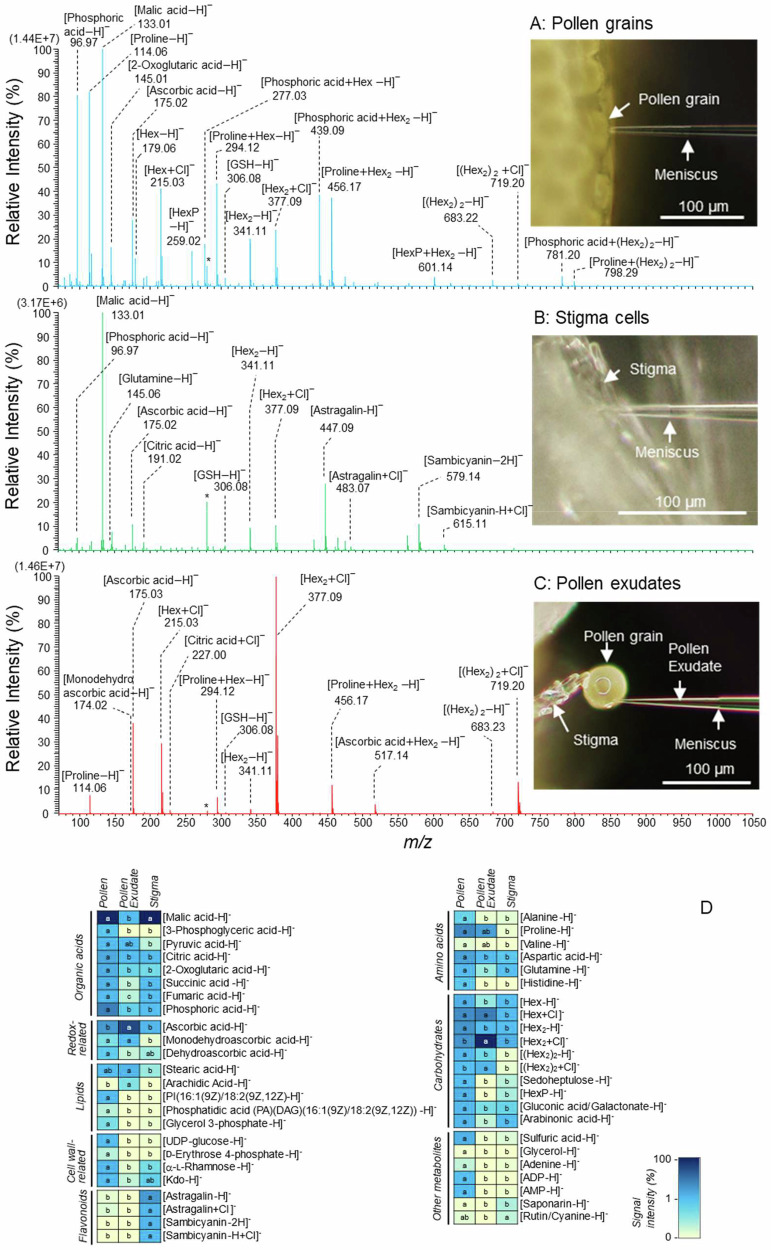
Site-specific metabolite profiling in pollen grains, stigma cells, and pollen exudates using picoPPESI-MS. PicoPPESI negative ion mode mass spectra obtained directly from single mature pollen grains prior to anther dehiscence (**A**), stigma cells (**B**), and pollen exudates prior to the pollen foot formation (**C**) under normal conditions. In (**D**) comparison of metabolites level in the deep blue (max value)-to yellow cream (minimum value) heatmap in the corresponding fluids in rice. Pollination was artificial, and picoPPESI analysis was conducted (see Methods). In (**B**) glycosylated flavonoid signals, astragalin (Kaempferol-3-O-glucoside) and sambicyanin (Cyanidin 3-xyloglucoside)-related signals were identified by conducting picoPPESI-MS/MS and -MS/MS/MS (see Fig. S4). The mass spectra in (**A**–**C**) are representative of the repeated measurements on 7–11 pollen grains from 4 to 5 independent plants. Different letters indicate significant differences as determined using a Tukey test: *P* < 0.05.

The picoPPESI-MS negative mode mass spectra show that many metabolite signals including cluster ions, that are assigned to organic acids, redox-related metabolites, lipids, amino acids, cell wall-related metabolites, glycosylated flavonoids, and carbohydrates have been detected in the exudates, mature pollen grains, and stigma, showing spatial variation in metabolites with high reproducibility (Fig. 3 and Supplementary Data 2). Mature pollen grains attached to the anthers just before anther dehiscence were richly contained numerous metabolites with the accumulation of organic acids, ascorbic acid, cell wall-related metabolites, amino acids (particularly proline), sugars (hexose and hexose2), hexose phosphate, glutathione, hydrogen sulphate, glycerol, adenine, ADP, and AMP (Fig. 3A, D, and Supplementary Data 2).

In stigma cells, four strong unknown signals at *m/z* 447.09, 483.07, 579.14, and 615.11 have been detected together with relatively smaller content of ascorbic acid and monodehydroascorbic acid, fatty acids, organic acids, amino acids, wall-related metabolites, and hexose2, compared with pollen grains (Fig. 3B and Supplementary Data 2). Furthermore, negative mode picoPPESI-MS/MS (and picoPPESI-MS/MS/MS) analysis was conducted with these four unidentified signals and compared with those of standard chemicals, Astragalin and Sambicyanin purchased, as well as the library information when it was available (see ‘Materials and Methods’). The observed fragmentation patterns from the precursor ions [Astragalin-H]^−^ and [Sambicyanin-2H]^−^ were both consistent with those well accepted for carbohydrates in negative ion mode (Fig. S4.1–7), known as *cross-ring cleavages*^26^. Besides, the well-known fragmentations for this family of compounds, involving H rearrangement to rationalise the loss of H_2_O during MS/MS analysis^26–29^ have also been observed (Fig. S4.1–7).

In contrast to the deprotonated ions, [M  −  H]^−^, no data related to chloride adducts for glycosylated flavonoid ions [M + Cl]^−^ fragmentations were available in the literature. When negative mode picoPPESI-MS/MS spectra were collected with the solutions of each standard glycosylated flavonoid in the presence of Cl anions, it has been observed that for Astragalin, the precursor ion, [Astragalin + Cl]^−^ at *m/z* 483.07 (Fig. S4.4) did not show the ion corresponding to the species formed by losing HCl, [Astragalin − H]^−^ (*m/z* 447.09 in Fig. S4.1). Similarly, in the Sambicyanin spectrum from the precursor ion [M + Cl]^−^, the fragment at *m/z* 579.13 as the species [Sambicyanin − 2H]^−^ because of HCl loss was not observed (Fig. S4.5). Both facts are indicating that the fragments generated in negative mode picoPPESI-MS/MS would keep Cl moiety in their structure (Figs. S4.4 and S4.5), not consistent with the general behaviour described for fragmentation of [M + Cl]^−^ with M carbohydrate^26–29^. The analysis shows that the fragmentation patterns from two precursor signals in the tissue extract assigned as [Astragalin + Cl]^−^ and [Sambicyanin-H + Cl]^-^ strikingly matched with the MS/MS spectra obtained from each standard solution. Therefore, the fragmentation schemes shown in Figs. S4.6 and S4.7 could be the most probable, and hence, putative molecular structures of neutral fragments based on the product ions detected have been illustrated in Fig. S4.8. Therefore, the picoPPESI-MS/MS and -MS/MS/MS analyses show that these four strong glycosylated flavonoids-related signals (detected as deprotonated ion, [M  −  H]^−^ and chloride adduct ion, [M + Cl]^−^ species) have been identified as astragalin (Kaempferol-3-O-glucoside) (i.e. *m/z* 447.09 and 483.07 were [Astragalin − H]^−^ and [Astragalin + Cl]^−^, respectively) and sambicyanin (Cyanidin 3-xyloglucoside) (i.e. *m/z* 579.14 and 615.11 were [Sambicyanin − 2H]^−^ and [Sambicyanin − H + Cl]^−^, respectively) (Figs. 3B, D and S4.1–4.8).

In contrast, the exudates were shown to contain sugars (hexose and hexose2), ascorbate, monodehydroascorbate, glutathione, proline, and saturated fatty acids, such as stearic acid and arachidic acid have been detected as major signals (Fig. 3C, D, and Supplementary Data 2). Strong chloride adduct ions were frequently observed for both hexose and hexose2 and their cluster ions in the exudates, contrastingly different from the cellular fluids from pollen grains and stigma (Fig. 3A, B, and D, and Supplementary Data 2).

## Discussion

In this study, we revisited the overlooked phenomenon, pollen exudation^6^ at early post-pollination in Gramineae (grass family) crops by performing picoPPESI-MS-based on-site metabolomics in the picolitre exudates directly collected from intact rice plants (Fig. S2 and Supplementary Movie 3). Consequently, we have discovered that rice pollen grains showed side-to-side rocking and winding motion subsequently after pollen exudation accompanied with a reduction in *V*_*pollen*_, behaving like *roly-poly toy* on the lubricant exudates aggregated on the stigma cells (Figs. 1B, 2 and S3, see Supplementary Movie 2). It has been strongly suggested that pollen exudation, followed by the motion, would allow each pollen grain to mechanically self-position on the lubricant exudates accumulated in the stigmatic apoplast, resulting in rapid pollen adhesion and germination (Fig. 2C–E and S3, see Supplementary Movies 1 and 2). Therefore, the pollen behaviours during post-pollination observed in rice might contribute to the rapidity of self-pollination, which is systematically different from general pollination pattern^6,8,9,30^ that lacks such considerable pollen exudation concomitant with a reduction in *V*_*pollen*_ (see the example of *A. thaliana* in Fig. 1A for the comparison). The difference in *V*_*pollen*_ regulation would be attributed to the differences in water relations during pre- and post-pollination, depending on the pollen and stigma morphology, as discussed below. Considering the early reports^6,7^ and our findings obtained in this study, we propose that similar mechanism may broadly exist in the hydrating pollen grains of self-pollinated grass plants in nature and cross-breeding. From this perspective, it is anticipated that pollen exudation might be a trait in cultivated rice that has attained to achieve rapid self-pollination by selective breeding during long-term domestication. And hence, the pollen behaviour we report here in rice might be of biological significance in enabling the rapid self-pollination required for domesticated grass crops.

Most flowering plants form the pollen foot at the interface between pollen grains attached and stigma cells during post-pollination, which leads to pollen hydration^1,2,8^. In *A. thaliana*, pollen grains are partially hydrated, and after pollen capture the progressive increase in *V*_*pollen*_ during pollen hydration occurs with water uptake from the stigma papillae (Fig. 1A)^1,2^. Contrastingly, rice plants have hydrating pollen grains, maintaining turgor prior to anther dehiscence (Fig. 2G). Rice *V*_*pollen*_ increases due to the water entry at pollen swelling just after floret opening^31^, although shortly after pollen capture *V*_*pollen*_ continuously declined due to pollen exudation (Fig. 2F). Our results indicated that rice pollen exudation followed by *roly-poly toy*-like motion preceded to pollen adhesion (Fig. 2C–E), resulting in pollen hydration presumably due to the water flow from the stigma cells, which initiated at ca. 2 min after pollen capture (Fig. 2B–F). The amount of exudates was relatively large, equivalent to 14.7% ( = 100 - 85.3%) of initial *V*_*pollen*_ at pollen capture (Fig. 2F), agreeing with the previous report^8^. The data also shows that rice pollen exudation occurred in about 20 s after pollen capture to initiate a progressive reduction in *V*_*pollen*_ (Fig. 2C, H), consistent with the early observations^6,8,10^. While water uptake from the stigma during pollen exudation has been long assumed in grass crops^6,8,9^, this study illustrates that rice pollen grains are capable for inducing germination without any contact with stigma, at least in cv. Koshihikari (Figs. 2 and S1). Therefore, the pollination pattern observed in rice-like grass crops should be reconsidered from the viewpoint of plant water relations as a distinct system from the general model^1,2^.

The pollen hydrodynamics at pollination in angiosperms was first interpreted by Heslop-Harrison^8^. This interpretation was made in partially dehydrated rye pollen, and the water potential of pollen, Ψ_*poll*_ was defined as follows^8^, according to the reference introduced by Slatyer^32^.

1 $${{{\Psi }}}_{{poll}}={{{\Psi }}}_{m}+{{{\Psi }}}_{s}+{{{\Psi }}}_{p}$$

where the components, Ψ_*m*_, Ψ_*s*_, and Ψ_*p*_ are regarded as the matric potential arising from the imbibitional properties of the cytoplasmic colloids, osmotic potential (i.e. the negative value of osmotic pressure) attributable to solutes on or in the pollen grain, and the pressure potential (i.e. turgor) equatable with wall pressure. Heslop-Harrison used Eq. (1) misleadingly on several points, as described in Supplementary Discussion 1. In addition, Eq. (1) representing the water potential^32^ did not discriminate the apoplastic and protoplasmic components of the cells.

When recognising that the pollen grains also form the two compartments separated by plasma membranes, the water potentials of protoplast (Ψ_*w*_^poll (*pro*)^) and apoplast (Ψ_*w*_^poll (*apo*)^) in the grains can be independently described as:

2 $${{{\Psi }}}_{{w}}^{{{\rm{poll}}}({pro})}={{{\Psi }}}_{{s}}^{{{\rm{poll}}}({pro})}+{{{\Psi }}}_{{p}}^{{{\rm{poll}}}({pro})}$$

3 $${{{\Psi }}}_{{w}}^{{{{\rm{poll}}}}({apo})}={{{\Psi }}}_{{s}}^{{{{\rm{poll}}}}({apo})}+{{{\Psi }}}_{{m}}^{{{{\rm{poll}}}}({apo})}$$

where Ψ_*s*_^poll (*apo*)^ and Ψ_*m*_^poll (*apo*)^ represent the apoplastic osmotic potential and matric potential, respectively. In addition, Ψ_*w*_^poll (*pro*)^ can be equilibrated with the Ψ_*w*_^poll (*apo*)^, i.e., Ψ_*w*_^poll (*pro*)^ ≈ Ψ_*w*_^poll (*apo*)^^21,33^. When considering plant pollination, the total water potentials in the stigma papilla cells can also be defined individually in the same manner, and similar interpretation can be applied to the stigma cells (see Supplementary Discussion 1). During the early post-pollination, it is presumed that the physicochemical interaction(s) of Ψ_*m*_ and Ψ_*s*_ in the two apoplastic spaces in contact between each pollen grain and stigma cells would form considerable surface tension of exudate, creating adhesion force between the pollen grain and stigma cell surface.

Our picoPPESI-MS analysis revealed clear site-specific differences in metabolites between pollen grains, stigma, and exudates at rice pollination (Fig. 3 and Supplementary Data 2). Pollen exudates were shown to be composed of various solutes, such as Hex2, fatty acids, and redox-related metabolites (Fig. 3 and Supplementary Data 2). The strong signals for the Hex2-related cluster ions suggest that sucrose might be major in the concentrated exudates (Fig. 3C). The saturated fatty acids detected in the exudates might be used for synthesising triacylglycerol that restores hydraulic contact in the foot structure^34^ and likely facilitate signalling cascades at pollen-stigma recognition^1,2^. Except for these, hydrocarbon^11^ and pollen coat proteins^35^ might be present in the exudates. In the pollen grains at exudation, Ψ_*s*_^poll (*apo*)^ would decline due to the considerable solute accumulation in concomitant with dehydration, which might cause a loss of Ψ_*p*_^poll (*pro*)^, as reported in other systems^21,36^. When exudates reached to the receptive part of stigma (i.e. a part of stigmatic apoplast), the apoplastic osmotic potential in the stigma at the interface could decline due to the mixing with the exudates. The rapid exchange and mixing of the apoplastic solutions on the stigma surface should occur until the apoplastic water potentials in the stigma and pollen grains reach an equilibrium. Since the dynamic changes in apoplast water status in stigma cells should precede to pollen adhesion and hydration in rice, it can be pointed that the solutes detected in the exudates would play important roles as an osmotic signal and increase the fluid viscosity for adhesion, besides the molecular signalling at pollen hydration^1,2,34,37^.

In rice, an increase in *V*_*pollen*_ causes pollen swelling, resulting in anther dehiscence^17^. A reanalysis of pollen swelling in cv. ‘Akihikari’^31^ showed that artificial floret opening induces 15% increase in *V*_*pollen*_ (*n* = 15, 6–26% between 2 and 28 min), whereas the same extent of the inverse and continuous reduction in *V*_*pollen*_ has been observed during rice pollen exudation, reaching 85.3% of the initial *V*_*pollen*_ (Fig. 2F), consistent with the report in rye^8^. For pre-pollination, an increase in Ψ_*p*_^poll (*pro*)^ might occur at pollen swelling. Hence, these two contrasting events during pre- and post-pollination, pollen swelling and exudation, might be closely associated with dynamic changes in water status in the generative cells^38^. During pollen hydration, water potential gradient could be established, and the pollen grains would be more concentrated to decline the water potential of each grain adhered, so that the Ψ_*w*_^poll (*pro*)^ could be lower than stigma water potential. Future studies on plant pollination will need to be reconsidered from the viewpoint of plant water relations.

Our data also indicates that the unusual rocking motion occurred on the lubricant exudates to self-position on the stigma cells, forming ‘pollen foot’- like structure (Fig. 2C–E, see Supplementary Movie 1). In general, acceleration should be required for any movement of objects from the physical view. In the case of the *roly-poly toy* motion, the rotation should occur according to the gravitational acceleration by moving the centre of gravity in the sphere object^39^. The mature rice pollen grains at anther dehiscence are reported to have a polarity due to the amyloplast localisation^40^. While the exact cause(s) of the motion remain obscure, the observed side-to-side rocking and winding motion (Fig. S3C, D, and Supplementary Movie 2) has arisen that organelle rearrangement, such as amyloplast sedimentation, might have occurred in the grains prior to the motion during the early post-pollination. In effect, gravity-induced sedimentation of amyloplast in root tips^41^ is well known as a similar phenomenon. Furthermore, it should not be ignored that pollen grains in monocotyledons, including rice, have a single aperture (germ pore), whereas the majority of dicotyledons have three apertures^42^. The exact position of the pollen surface at the final position of the motion could not be determined in this study; however, it can be speculated that the pollen motion in rice might bring the germ pore to be close to stigma (see Fig. 1B). Making a detour observed in the motion analysis (see Fig. S3C, D) suggests that surface tension effect is also involved in the motion, generating frictional resistance to contribute to the adhesion of pollen grains. And thus, the motion found in rice pollen grains could be attributed to both gravitational and adhesive surface tension.

In addition, the contact angle between the exudate and stigma surface at pollen adhesion was 74° on average (Fig. 2K), indicating middle to high wettability. This refers to the presence of larger adhesive forces between the pollen grains and stigmatic surface than cohesive forces at pollen adhesion, as above-pointed from the viewpoint of plant water relations. And, this would be attributed to interaction behaviour of matric potential and osmotic potential in both apoplastic spaces. In stigma cells, picoPPESI-MS, -MS/MS, and -MS/MS/MS analyses revealed the active accumulation of two glycosylated flavonoids, astragalin (Kaempferol-3-O-glucoside) and sambicyanin (Cyanidin-3-xyloglucoside) in the cellular fluids of stigma cells (see Results and Figs. 3B and S4). To the best of our knowledge, this is the first report to characterise chloride adducts of these glycosylated flavonoids by MS/MS analysis (Figs. S4.1 to S4.8). These endogenous effective antioxidants most likely prevent reactive oxygen and nitrogen species to mediate cellular redox state in stigma papillae^43^. Since the microcapillary tip is assumed to be located in the vacuoles of stigma cells during the pressure probe operation^44^, these glycosylated flavonoids would be present in the vacuoles. Because external flavonoid (Kaempferol) treatment is capable of cancelling self-incompatible response to cause self-pollination in *Brassica*^45^, the greater accumulation of astragalin and sambicyanin in stigma cells (Fig. 3B, D) would mediate rapid self-pollination in rice with allogamy of anemophilous flowers^46^. More recently, self-incompatibility triggers stigmatic ROS production that determines the rejection of incompatible pollen, illustrating the importance of redox regulation on compatibility^47^.

Successful pollen adhesion, subsequent pollen hydration and greater number of germinated grains would be the prerequisites for higher spikelet fertility. Currently, establishing steady production of grass plants as staple food is urgent in global food security under climate change. A decrease in the number of germinated rice pollen grains on the stigma that leads to spikelet sterility^16,48^ might be caused by the inactive motion due to the altered exudate composition originating from heat-induced changes in pollen water relations and metabolisms^4^. Whether heat-tolerant rice cultivars have similar exudation patterns remains questionable. These cultivars might have an ability for germinating without relying on stigma water, although the underlying mechanisms remain unknown. Much effort is needed to answer these questions. In addition to the major roles on foot formation and tube growth of lipids identified in the exudates, lipids in rice exudates may have another impact on allergic inflammation^49^. It is anticipated that pollen exudation might aggravate pollen allergy symptoms, which is predicted to be more serious under the influence of climate change^50^. In this study, we report a unique pollen behaviour during the early post-pollination, identifying the chemical composition of pollen exudates by performing picoPPESI-MS analysis. It has been unveiled that picolitre pollen exudation, followed by the rocking motion, allowed each pollen grain to self-position on the exudates accumulated in stigma, leading to the pollen adhesion and germination to contribute to the rapid pollination in rice. And, pollen grains in self-pollinated grass crops might have similar *roly-poly toy* dynamics. Further work from this perspective at cell level might contribute to the development of grass breeding under climate change, in addition to further understanding of heat-induced spikelet sterility.

## Methods

### Plant materials

The growth-chamber experiment was conducted at the Kyushu Okinawa Agricultural Research Center, National Agriculture and Food Research Organisation, in Chikugo, Fukuoka, Japan. Seedlings of rice (*Oryza sativa* L. cv. ‘Koshihikari’) at 2 weeks old were transplanted into the plastic pots (diameter 7 cm, height 30 cm) containing a lowland paddy soil (Typic Endoaquepts), and a plant per pot was prepared by removing the tillers^51^. The pots were kept in an environmentally controlled walk-in growth chamber (K260B029-S01, Tsubuku Corporation Ltd, Kurume, Japan) with a photoperiod of 13/11 h day/night at 26/22 °C, 70/80% relative humidity, and 750 μ mol photons m^−2^ s^−1^ photosynthetically active radiation (PAR) set at the plant canopy to grow until they reached the mature stage of plants (40 days after heading; DAH). At maturation, spikelet fertility in Koshihikari was determined.

### Microscopic observations

The anthers in dehiscence with ungerminated mature pollen grains were gently removed with a precision scissors. The top half of the lemma of pre-flowered spikelet, located at the first to third primary rachis branches, counted from the top of panicle, was gently removed under humid conditions. And immediately, cross-pollination was artificially conducted in the attached spikelet by tapping the anthers with fingers before self-pollination occurred in the spikelet. During the microscopic observations, environmental conditions were set at 26 °C and 70% RH under 1011.2 ± 4.5 (mean ± SD, *n* = 32) hPa atmospheric pressure. The atmospheric pressure was obtained from the barometer set at position of 33 m above sea level in Saga weather station (33° 16.0’ N, 131° 18.2’ E), Japan Meteorological Agency, which was ~18.5 km away from the Kyushu Okinawa Agricultural Research Center. The pollen grains adhered onto the stigma were viewed throughout pollen germination with a digital microscope (KH-8700, HIROX Co. Ltd, Tokyo, Japan) (see Supplementary Movie 1). The anthers used were independently collected, and the numbers of pollen grains per anther were counted. *V*_*pollen*_ after pollen capture was determined, and the number of pollen grains in the anthers and adhered on the stigma were both counted. In some cases, ungerminated pollen grains in the dehiscing anthers were either dipped into the Type (A) microscope immersion oil (16482, Cargille Laboratories, NJ, USA) or placed onto the adhesive tape. And immediately, they were photographed over time with the same microscope (see Discussion, Fig. S1).

### Image analysis of rice pollination process

With the pollination process videotaped, the timing of each event (i.e. pollen capture, pollen exudation, pollen hydration, and germination) was recorded. For the time-course of changes in pollen diameter after pollination, the outline of each pollen grain in the JPEG images (frames) extracted with Adobe Premiere Elements 2021 from the video files was traced by using the ImageJ software (https://imagej.nih.gov/ij/). The pollen diameter was estimated, assuming that the grain was spherical. And, pollen grain volume, *V*_*pollen*_ was calculated according to *V* = 4/3πr^3^. Regarding the grain rotation during *roly-poly toy*-like motion, two frames that corresponded to the initial and final points during the unidirectional motion were extracted, and thereafter, the actual moving distance was determined. By assuming that pollen diameter holds constant during the motion, the angle of rotation was estimated with the distance determined. For the measurement of contact angle at pollen adhesion, the jpeg images extracted from 12 mpeg files, in which foot-like structure was observable, were extracted. And then, the contact angle established as the tangential angle of the liquid droplet (pollen exudate) with a solid stigmatic surface (see *θ* in Fig. 2E) was determined with ImageJ software. For the pollen motion analysis, the pixel coordinates corresponding to the center of pollen grains were determined every second by using ImageJ software.

### On-site cell and fluid metabolomics

At the initiation of flowering, potted rice plants were transferred from the growth chambers to the adjacent cell measurement room controlled under the identical conditions (see Fig. S1 in ref. ^20^). A pot containing plants at flowering was placed at the centre of a U-shaped vibration-free table in the measurement room^20^. The CPP system and plants were allowed to reach temperature equilibrium prior to the metabolomic analysis^20^. Artificial pollination was then similarly conducted as described above and pollen exudation was induced (see Results, Supplementary Movie 1). In CPP, quartz microcapillary of 1.0 mm in outer diameter and 0.7 mm in inner diameter was used, and the microcapillary tip was fabricated reproducibly with a laser micropipette puller (P-2000, Sutter Instrument Co., CA). The microcapillary tip prefilled with a 0.01% (v/v) ionic liquid/silicone oil mixture^19^ was produced by breaking open to be ca. 2 μm i.d. by fine collision against the surface of Styrofoam. The set-up of a CPP was same with the previous work^4,20^. Simply, each spikelet was gently fixed on the sample holder using tape and magnets (see Fig. S1 in ref. ^20^). A quartz microcapillary tip installed in the pipette holder was maintained at zero pressure. By using a Piezo manipulator (DC-3K, Märzhäuser Wetzlar, Germany) under the digital microscope, the tip position of fine quartz microcapillary was precisely controlled and approached to the exudates just released from the grain surface (Supplementary Movie 3). And thereafter, the tip was inserted into the exudates and then the fluids were quickly collected by depressurising the microcapillary by the aid of CPP^52^. In addition, the microcapillary tips were used to penetrate the pollen grains adjacent to the inner wall of the middle theca in the developing anthers and the stigmatic cells in the attached spikelet at the same stage, to independently collect picolitre fluids in those cells as comparisons. For the mature pollen grains, cell turgor was also determined with CPP according to the previous works^20,52^. To conduct the electrospray (ESI) volatilisation/ionisation process an internal electrode has been embedded into the CPP capillary holder in picoPPESI-MS. Immediately after each fluid collection, the probe tip was rotated and oriented toward the entrance of an Orbitrap mass spectrometer (Q-Exactive, ThermoFisher Scientific Inc., MA, USA), and the tip was subsequently charged at −4 kV using a high-voltage generator (AKTB-05k1PN/S, Touwa Keisoku Corp., Tokyo, Japan). The Orbitrap mass spectrometer (Q-Exactive) was calibrated with the Pierce ESI negative ion calibration solution (ThermoFisher Scientific Inc.) prior to the experiment on each measurement day. On-site picolitre fluid metabolomics was conducted in each sample without any dilution by modifying the previous method^4^ using picoPPESI-MS^19^ placed under the same environmental conditions^20^. The measurement duration of picoPPESI-MS analysis was within a few minutes per shot. Although humid conditions induced anther dehiscence^17^, no septum rupture occurred during the above pollen fluid collection. All manipulations were conducted under the same digital microscope. Exact monoisotopic *m/z* values for all the peaks on the mass spectra acquired were extracted using the Qual Browser application in the Thermo Xcalibur software (ThermoFisher Scientific). Metabolites were identified from the theoretical masses of candidate metabolites in either the METLIN online metabolomics database (https://metlin.scripps.edu/index.php) or the Thing Metabolome Repository (http://metabolites.in/things/), allowing differences of <5  ppm. With the MS spectra collected using picoPPESI-MS, a deep blue (max value) to yellow cream (minimum value) heatmap was drawn using Microsoft Excel. The mass spectra reported here are representative of the repeated measurements on 8–11 pollen grains from 4 to 5 independent plants in each treatment.

### Identification of astragalin and sambicyanin in stigmas

MS/MS (and MS/MS/MS) analysis for four unidentified signals at *m/z* 449.09, 483.07, 579.14, and 615.11 observed in stigma samples was conducted on the crude stigma extracts from field-grown Koshihikari plants cultivated in Ehime University in 2023. Samples (*n* = 3) were collected in the field between August 18 and 20, 2023, to be stored at −80 °C until analysis. The pooled stigmas from six spikelets attached to the corresponding position in a panicle were collected using forceps, and tissue homogenate was then mixed with 50% (v/v) water/methanol and sonicated. After centrifugation for 10 min at 2000 × *g* at 4 °C, the supernatant (i.e. crude stigma tissue extract) was used for tissue MS analyses. Collision-induced dissociation (CID) tandem MS analysis was then performed in negative ion mode using the Orbitrap MS (Orbitrap Elite, ThermoFisher Scientific Inc., MA, USA) coupled with the picoPPESI system. Prior to the experiment, Orbitrap Elite was similarly calibrated as described above. The MS/MS and MS/MS/MS scan spectra were acquired with the instrumental settings of 100 ms as maximum injection time, inlet ion transfer tube temperature of 275 °C, resolution of 60,000, and AGC value of 5 × 10^4^. The chemical assignments were based on the fingerprint of precursor ions compared with the standard chemical patterns from either the library information or the MS/MS patterns measured with the standards purchased (see Fig. S4). In some cases, the formula prediction from the picoPPESI-MS/MS was manually conducted (Fig. S4). All the standard chemicals and organic solvents used in the experiments were LC/MS grade purchased from FUJIFILM Wako Pure Chemical Corp. (Osaka, Japan). Standard chemicals, astragalin and sambicyanin, were HPLC grade purchased from Merck. Ultrapure water of 18.2 MΩ cm^−1^ was used throughout the experiment.

### Pollen exudation test

To identify the source of pollen exudates, mature pollen grains just before anther dehiscence were collected and immediately either dipped in the immersion oil put on the slide glass or placed onto the adhesive tape (see each inset in Fig. S1), and they were viewed under the digital microscope and photographed for 3 min after pollen capture.

### Spikelet fertility

After harvest, panicles were dried at 30 °C for 3 days. Thereafter, the number of mature seeds and empty caryopses was counted. The values reported for spikelet fertility represent the means of seven independent plants.

### Statistics and reproducibility

The statistical significance of site-specific differences in metabolites detected by picoPPESI-MS analysis was performed using Tukey’s test in JMP (version 12.1.0; SAS Institute Inc., Cary, NC, USA) and the generation of a heatmap was conducted using Microsoft Excel 2019. A *P* value less than 0.05 was considered statistically significant. For microscopic observations, pollen grains in focus taken in the video files were only used for further analyses (pollen size and event-based duration measurements). Other pollen grain samples out of focus were excluded from the analyses. During the direct turgor determination in intact cells and picolitre fluids (exudates) extraction, samples impaled with microcapillary tips that lacked hydraulic connection (i.e. tip plugging) were excluded from turgor measurements and picoPPESI-MS analysis following standard practice. Except for these, no data were excluded in other data collections. We used seven independent in-focus biological samples in characterising the changes in pollen diameter after pollen capture. For the determination of initiation time of each event (pollen exudation and *roly-poly toy*-like motion) after pollen capture, 29 and 26 replicates were used, respectively. For the determinations of pollen turgor, seven replicates were used. For the measurements of rocking motion-related parameters (i.e. pollen moving distance, and angle rotated during the rocking motion) and contact angle at pollen adhesion, 22 and 12 replicates were used, respectively. For picoPPESI-MS analysis, 7–11 replicates obtained from 4 to 5 independent plants were used. For chemical identifications in MS/MS and MS/MS/MS analyses for stigmatic unknown signals, three biological replications in field-grown rice plants were used to collect the tissue extracts. All samples were randomly collected. Blinding was not applied in this study.

### Reporting summary

Further information on research design is available in the Nature Portfolio Reporting Summary linked to this article.

## Supplementary information

Supplementary Information

Description of Additional Supplementary Files

Supplementary Data 1

Supplementary Data 2

Supplementary Movie 1

Supplementary Movie 2

Supplementary Movie 3

Reporting summary

## Data Availability

All the data generated in this study are available in the paper and Supplementary Information (Supplementary Figs. S1–S4, and Supplementary Movies 1–3) and Supplementary Data 1 and 2. The movie of pollen exudation followed by *roly-poly toy* motion in rice pollen grains is provided in Supplementary Movies 1 and 2. Pollen exudate collection using a cell pressure probe is provided in Supplementary Movie 3. Source data for figures and the statement in Results can be found in Supplementary Data 1.
