# Supplementary material for: ‘Roly-poly toy’ motion during pollen exudation promotes rapid pollen adhesion in rice
Source: Commun Biol. 2025 Apr 18;8:608. doi: 10.1038/s42003-025-08018-7 (PMC12008421; doi:10.1038/s42003-025-08018-7)
Supplement: Supplementary file 1 — Supplementary Information [file 42003_2025_8018_MOESM1_ESM.pdf]

Supplementary Information for

'Roly-poly toy' motion during pollen exudation promotes rapid pollen adhesion in rice

Hiroshi Wada, Yuto Hatakeyama, Rosa Erra-Balsells, Takumi Muneta, Hiroshi Nonami, Hikari Ueda, Yoko Yamaga-Hatakeyama, Naoya Miyashita, and Takuya Araki

Content the supplementary information

Supplementary Discussion 1: *'Reinterpretation on water relations of pollination process in grass plants'* and *'Theory of cell water relations during pollination in angiosperm'*.. 2

Supplementary Figure S1-S4 5

Supplementary References ....…. 16

Supplementary Discussion 1

Reinterpretation on water relations of pollination process in grass plants

In angiosperms, the hydrodynamics of pollen at pollination was first interpreted by Heslop-Harrison[1]. He described pollination process composed of pollen hydration and exudation in rye and provided an interpretation based on the plant water relations. In this report, he defined the water potential of pollen, Ψ_{poll} according to the reference[2].

$\Psi_{poll} = \Psi_m + \Psi_s + \Psi_p$ Eq. (1),

where the components, Ψ_m, Ψ_s, and Ψ_p are regarded as the matric potential arising from the imbibitional properties of the cytoplasmic colloids, osmotic potential attributable to solutes on or in the pollen grain, and the pressure potential (i.e., turgor) equatable with wall pressure.

The Eq. (1) representing the water potential introduced by Slatyer[2] did not discriminate the apoplastic and protoplastic components of the plant cells. Heslop-Harrison attempted to use the equation in the report[1], and consequently he misled the following points. First, they defined that Ψ_m is matric potential related to imbibitional properties of the cytoplasmic colloids as a component of water potential in the cells, i.e., pollen grains (vegetative cells). There would be many colloid-like structures in the pollen grains; however, those colloids make any contribution to total water potential in the plant cells, as shown by Passioura[3]. Heslop-Harrison seemed to misunderstand roles of cytoplasmic colloids on the matric potential in plant cells. Second, apoplastic and cytosolic solutes in pollen grains (solutes on or in the pollen grain) could be different and they cannot be regarded as the same parameter. Third, he postulated that Ψ_p is negative. However, it is most unlikely that cross-pollinated rye pollen grains exhibited negative Ψ_p, even though the grains had been desiccated at pollen capture (see Fig. 1A in Ref[1]). Fourthly, it appears that pollen grains are composed of apoplastic and protoplastic components. Apoplastic space in pollen grains at pollen dispersal should be exposed to air, and then dehydration likely occurs (i.e., under tension). Therefore, matric potential in apoplast should be negative, not positive. Regarding the pollination process, it is essential to define the total water potential in each component, as we will define below.

Theory of cell water relations during pollination in angiosperm

Here we can recognize that pollen grains (composed of mostly, vegetative cells) form the two compartments separated by plasma membranes, the water potentials of protoplast ($\Psi_w^{\mathrm{poll}\ (pro)}$) and apoplast ($\Psi_w^{\mathrm{poll}\ (apo)}$) can be independently described as:

$$\Psi_w^{\mathrm{poll}\ (pro)} = \Psi_s^{\mathrm{poll}\ (pro)} + \Psi_p^{\mathrm{poll}\ (pro)} \quad \text{Eq. (2)}$$

$$\Psi_w^{\mathrm{poll}\ (apo)} = \Psi_s^{\mathrm{pollen}\ (apo)} + \Psi_m^{\mathrm{poll}\ (apo)} \quad \text{Eq. (3),}$$

where $\Psi_s^{\mathrm{pollen}\ (apo)}$ and $\Psi_m^{\mathrm{poll}\ (apo)}$ represent the apoplastic osmotic potential and matric potential, respectively. And, protoplast water potential can be equilibrated with the apoplastic water potential, i.e. $\Psi_w^{\mathrm{poll}\ (pro)} \approx \Psi_w^{\mathrm{poll}\ (apo)}$[4,5].

Similar interpretation can be applied to the stigma papillae. The water potential of the protoplast ($\Psi_w^{\mathrm{stig}\ (pro)}$) and apoplast ($\Psi_w^{\mathrm{stig}\ (apo)}$) in stigma papillae can be described separately,

$$\Psi_w^{\mathrm{stig}\ (pro)} = \Psi_s^{\mathrm{stig}\ (pro)} + \Psi_p^{\mathrm{stig}\ (pro)} \quad \text{Eq. (4)}$$

$$\Psi_w^{\mathrm{stig}\ (apo)} = \Psi_s^{\mathrm{stig}\ (apo)} + \Psi_m^{\mathrm{stig}\ (apo)} \quad \text{Eq. (5).}$$

$\Psi_{\mathrm{s}}^{\mathrm{stig}\ (apo)}$ and $\Psi_m^{\mathrm{stig}\ (apo)}$ are regarded as the apoplastic osmotic potential and matric potential in stigma papillae, respectively. Likewise in stigma papillae, $\Psi_w^{\mathrm{stig}\ (pro)} \approx \Psi_w^{\mathrm{stig}\ (apo)}$ can be assumed.

Aside from the misinterpretation on pollen water relations, Heslop-Harrison[1] also described on the pollen exudation[6] (see Fig. 1B in Ref[1]). However, his interpretation was only based on pollen hydration followed by pollen exudation with bench-top dried rye pollen. There was essentially no interpretation in the process starting from pollen exudation in rye. In hydrating rice pollen grains, we have also observed that pollen exudation followed by 'roly-poly toy'-like motion preceded all other events including pollen adhesion and pollen hydration, where stigmatic water flow was found to occur. Furthermore, pollen turgor at pollen dispersal was positive throughout swelling (see Results). While the differences in the sequence of two pollination processes might be attributed to the pattern of cross- and self-pollinations, the early interpretation should be corrected from the viewpoint of plant water relations.

Our metabolome data illustrates that rice exudates eluted were composed of numerous solutes (see Fig. 3D and Table S1) and they were much concentrated than originally expected. Therefore, pollen exudation process would make $\Psi_s^{poll\ (apo)}$ more negative, which might induce a decrease in $\Psi_p^{poll\ (pro)}$[5,7]. When the exudates reached at the receptive part of the papillae (i.e., a part of stigmatic apoplast) throughout the pollen apoplast (exine) to initiate the formation of a foot-like structure, it is assumed that the reduced $\Psi_s^{poll\ (apo)}$ would influence $\Psi_s^{stig\ (apo)}$ at the stigmatic apoplast, which might trigger molecular signaling. Rapid exchange and mixing of apoplastic solution might occur until $\Psi_w^{stig\ (apo)} = \Psi_w^{poll\ (apo)}$. Under such a circumstance, surface tension of exudates would be formed due to the physicochemical interaction(s) of Ψ_m and Ψ_s in the two apoplastic spaces in contact between each pollen grain and stigma cells, generating subtantial adhesion force at the interface (see Discussion). The chemical compounds detected in the exudates could contribute to the rapid foot formation at the receptive part of the stigma cells, leading to pollen adhesion. Similar pollen exudation followed by ‘roly-poly toy’ motion may commonly exist in grass plants (see Discussion). Taken these together, this reinterpretation should be considered in the future research.

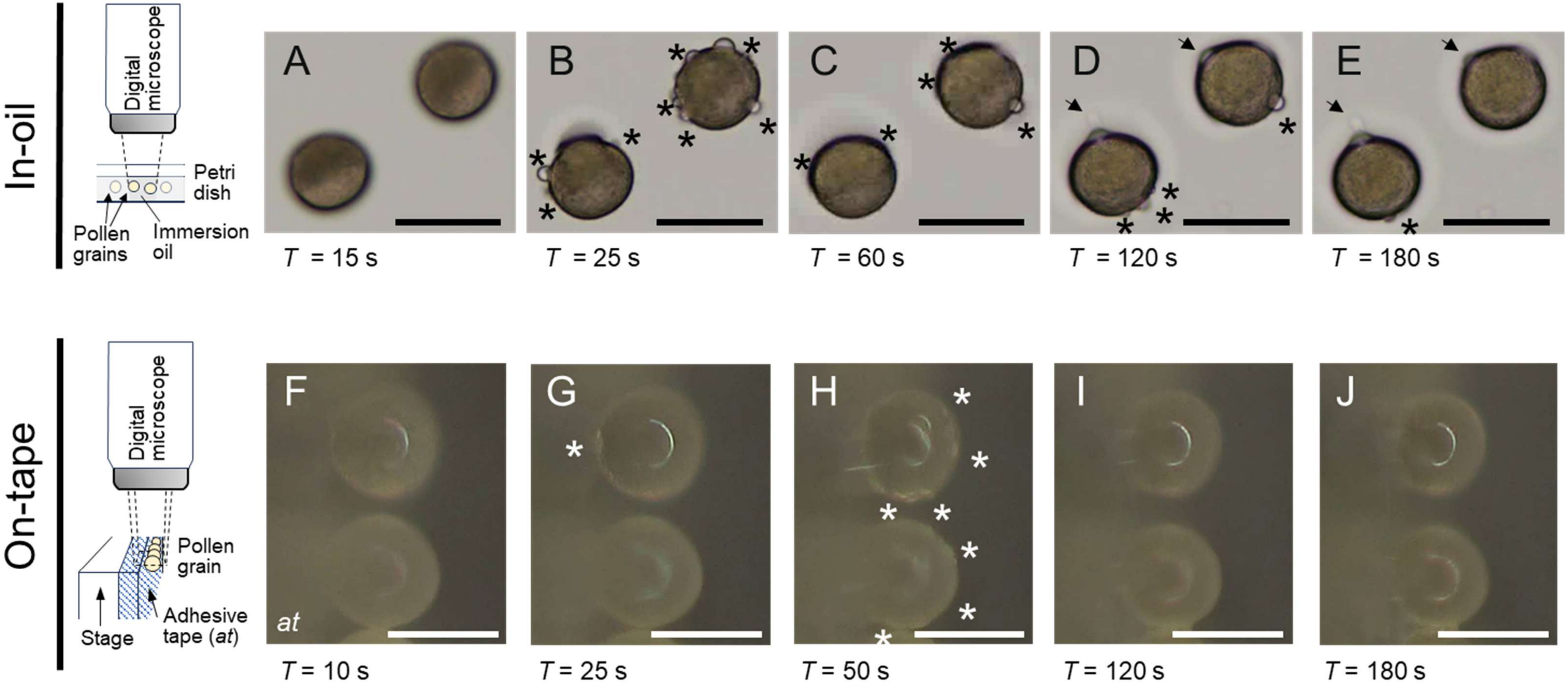

Figure S1. Rice (cv. 'Koshihikari') pollen grain images taken at in-oil (A-E) and on-tape (F-J) experiments. Time-course images of pollen grains at 15 s, 25 s, 60 s, 120 s, and 180 s after dipping into the microscopic immersion oil (A-E) and at 10 s, 25 s, 50 s, 120 s, and 180 s after placing onto the adhesive tape (F-J). After dipping into the oil, pollen exudation occurs in ca. 20 s in each pollen grain (B) and each exudate merged to cover the grain surface until 60 s (C). The asterisks and arrows indicate exudates being released from the pollen grains and growing pollen tube, respectively. In F-J, '*at*' indicates adhesive tape. Note that rice pollen grains exhibited polarity prior to pollen exudation (see A). Bars in A-J show 50 μm.

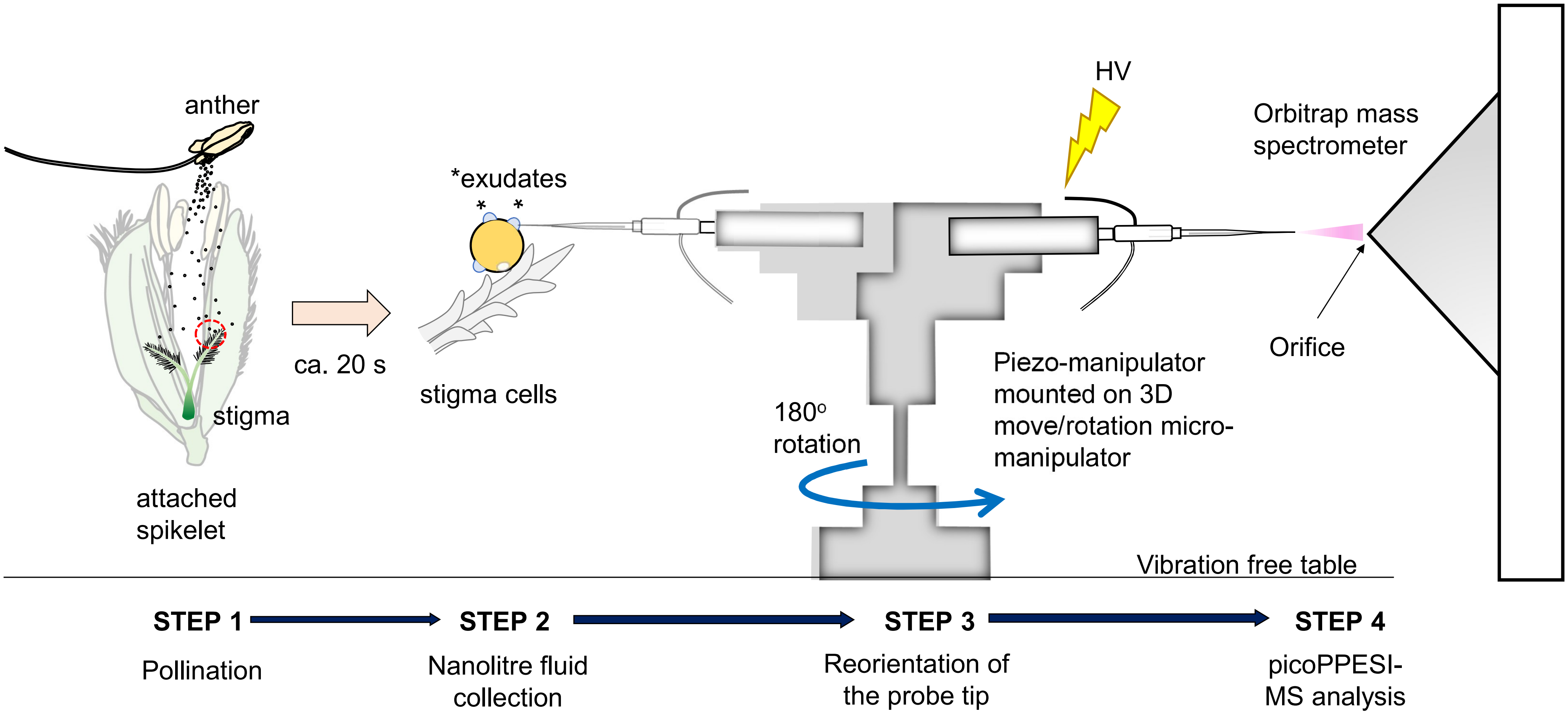

Figure S2. Illustrated workflow of artificial self-pollination, instantly followed by on-site real-time picoPPESI-MS analysis in picolitre fluids exudated from pollen grains. Also, see Supplementary Movie 2. The cartoon was generated with a partial modification from Wada et al. (2021)[24].

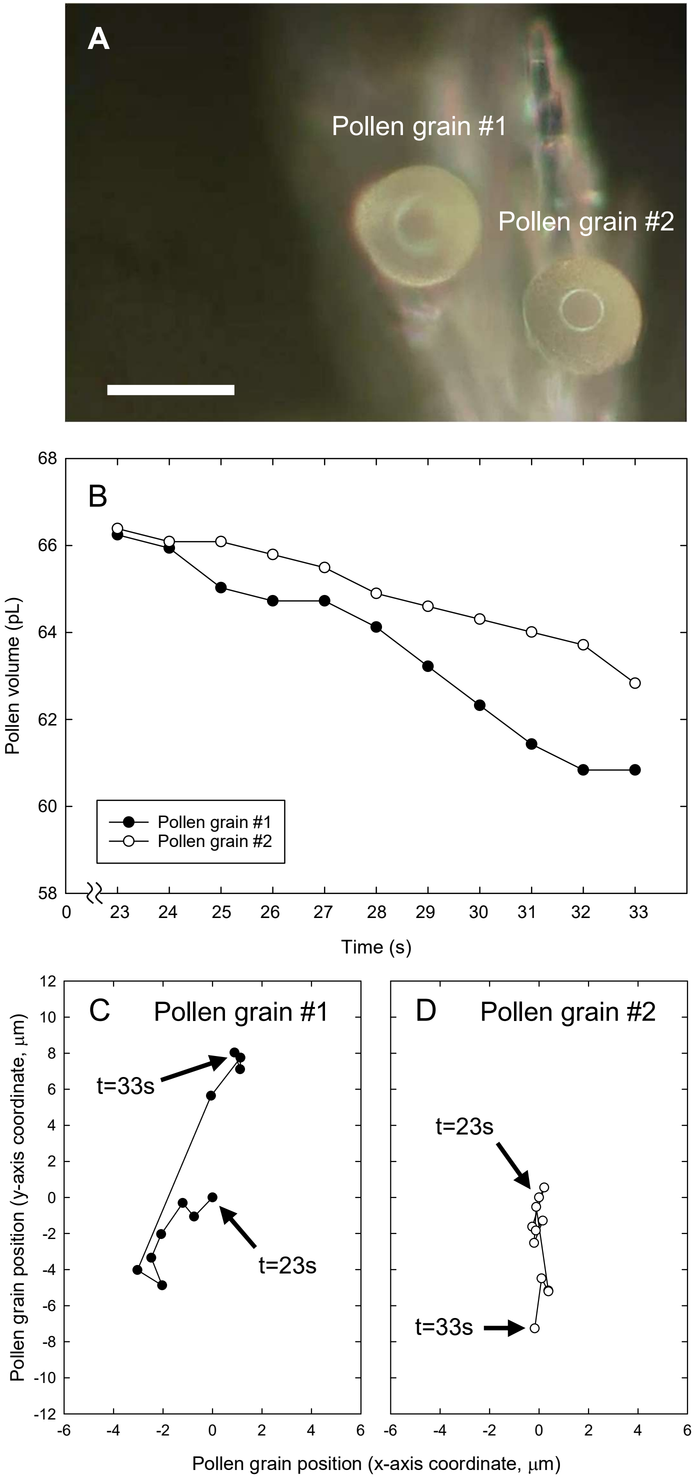

Figure S3. (A) The image of two pollen grains, Pollen grain #1 and #2 at t=23 s after pollen capture, corresponding to the beginning of the *roly-poly toy*-like motion. Note that the image was taken from the top of the pollen grains. (B) Time-course changes in the volume of each pollen grain during the motion. (C and D) Positional changes of each pollen grain every second between t=23 s and 33 s after pollen capture, where different *roly-poly toy*-like motions occurred. Also see Supplementary Movie 3. Bar in A shows 50 μm.

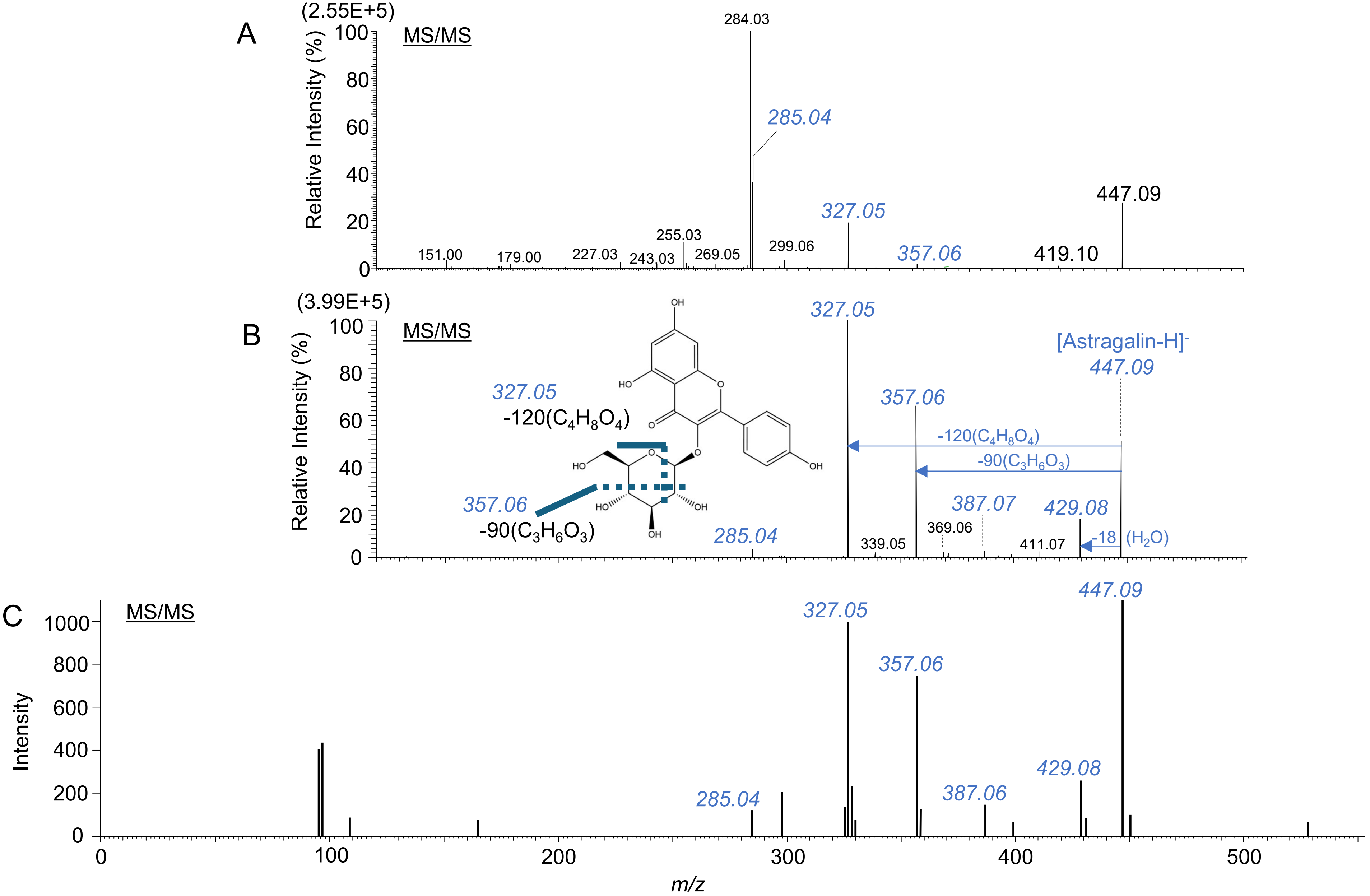

Figure S4.1. (A) PicoPPESI-MS/MS negative ion mode spectrum of a putative Astragalin-related peak ($[M-H]^-$, *m/z* 447.09) obtained from 1mM Astragalin (Kaempferol 3-β-D-glucopyranoside). Precursor ion *m/z* 447.0925; selector gate range *m/z* 446.64-447.54. Fragmentation was induced with a 28 % normalized collision energy. (B) PicoPPESI-MS/MS negative ion mode spectrum of a putative Astragalin-related peak ($[M-H]^-$, *m/z* 447.09) obtained from the rice stigma tissue extracts (see Materials and Methods). Precursor ion *m/z* 447.0925; selector gate range *m/z* 446.64-447.54. Fragmentation was induced with a 25 % normalized collision energy. The three signals with *m/z* shown in blue in A matched with B. (C) MS/MS spectrum for Astragalin from Thing Metabolome Repository data bank (http://metabolites.in/things/peak/221023/neg/4093). The five signals with *m/z* shown in blue in B matched with C. According to the MS/MS spectrum in A, the precursor ion was identified to be $[Astragalin-H]^-$ (see Fig. S4.2 for the chemical identification).

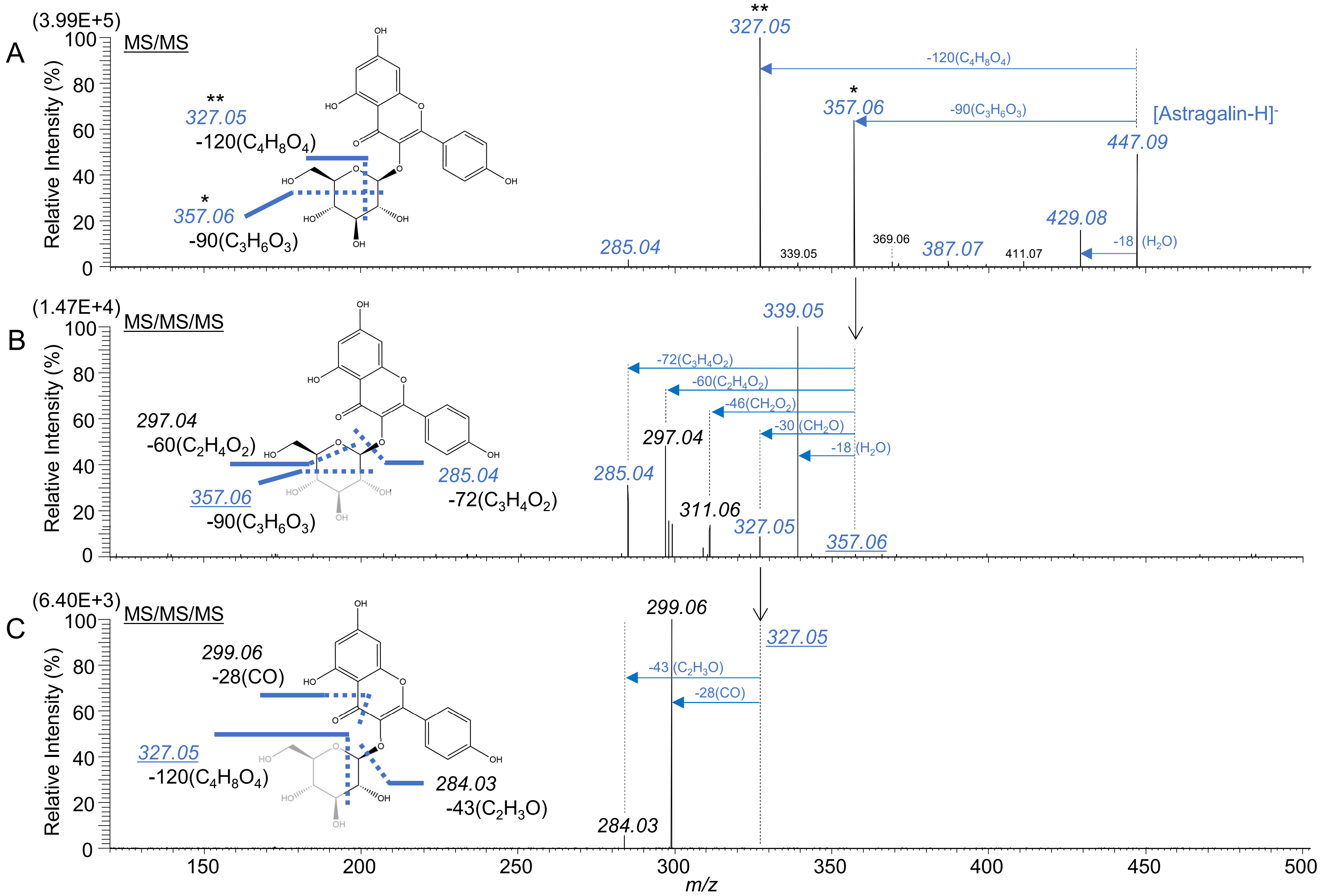

Figure S4.2. (A) PicoPPESI-MS/MS negative ion mode spectrum of a putative Astragalin-related peak ([M-H]$^-$, *m/z* 447.09) obtained from the rice stigma tissue extracts (see Materials and Methods). Precursor ion *m/z* 447.0925; selector gate range *m/z* 446.64-447.54. Fragmentation was induced with a 25 % normalized collision energy. (B) picoPPESI-MS/MS/MS negative ion mode spectrum of *m/z* 357.06 (*) generated from fragmentation of a putative Astragalin-associated *m/z* 447.09 signal. Precursor ion *m/z* 357.06; selector gate range *m/z* 356.61-357.51. (C) picoPPESI-MS/MS/MS negative ion mode spectrum of *m/z* 327.05 (**) generated from fragmentation of another putative Astragalin-associated *m/z* 447.09 signal. Precursor ion *m/z* 327.05; selector gate range *m/z* 326.60-327.50. Fragmentation in (B) and (C) was both induced with a 40% collision energy. According to the MS/MS and MS/MS/MS spectra in A-C, together with Fig. S4.1, the precursor ion observed at *m/z* 447.09 was identified as [Astragalin-H]$^-$.

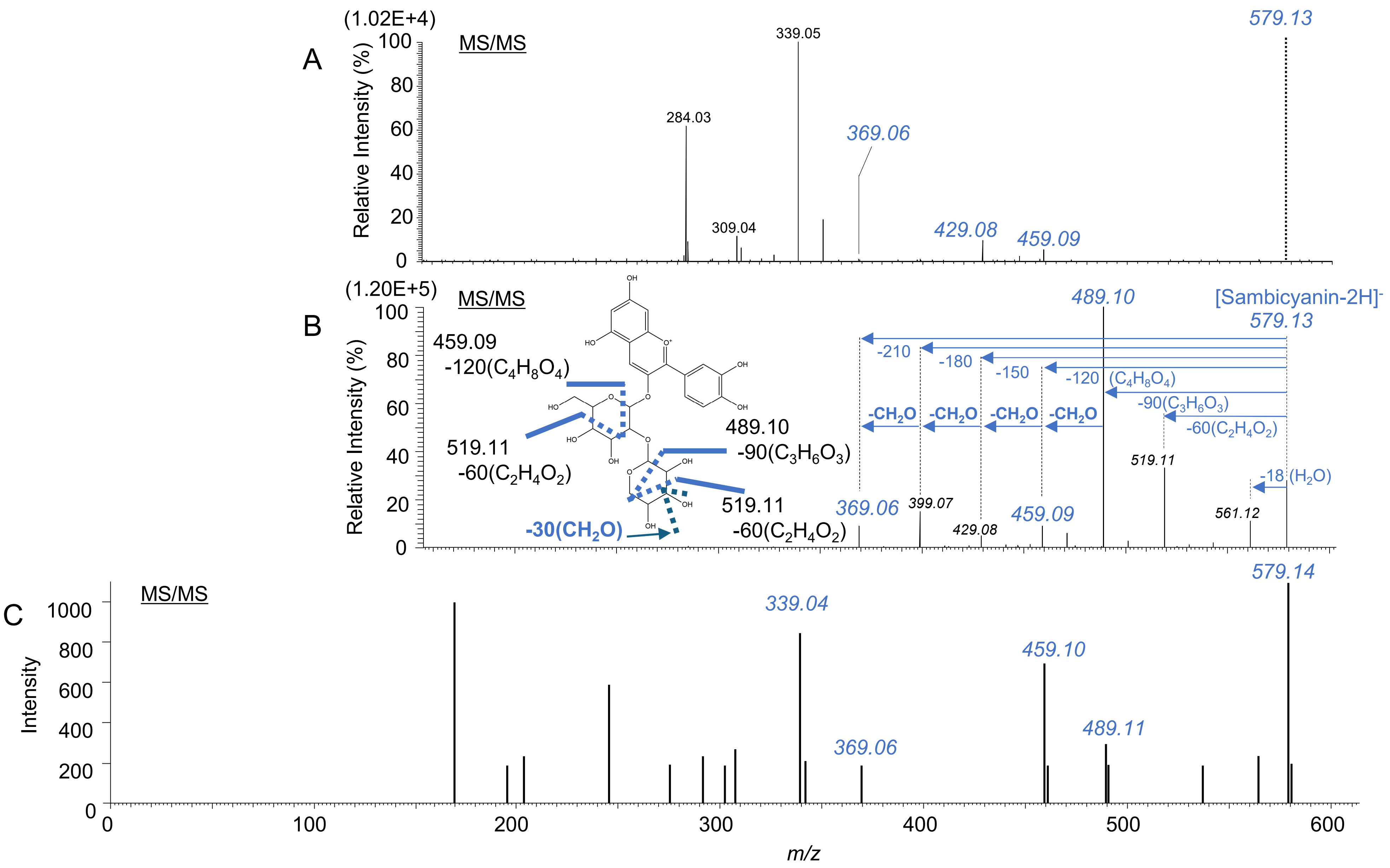

Figure S4.3. (A) PicoPPESI-MS/MS negative ion mode spectrum of a putative Sambicyanin-related peak ([M-2H]$^-$, *m/z* 579.13) obtained from 0.714mM Sambicyanin (Cyanidin 3-sambubioside) chloride. Precursor ion *m/z* 579.1335; selector gate range *m/z* 578.68-579.58. Fragmentation was induced with a 30 % normalized collision energy. (B) PicoPPESI-MS/MS negative ion mode spectrum of a putative Sambicyanin-related peak ([M-2H]$^-$, *m/z* 579.13) obtained from the rice stigma tissue extracts (see Materials and Methods). Precursor ion *m/z* 579.1335; selector gate range *m/z* 578.68-579.58. Fragmentation was induced with 25 % normalized collision energy. The three signals with *m/z* shown in blue in A matched with B. (C) MS/MS spectrum for Sambicyanin ([M-2H]$^-$) from Thing Metabolome Repository data bank (http://metabolites.in/things/peak/221001/neg/7488). The three signals with *m/z* shown in B matched with C. According to the MS/MS spectrum, the precursor ion was identified as [Sambicyanin-2H]$^-$.

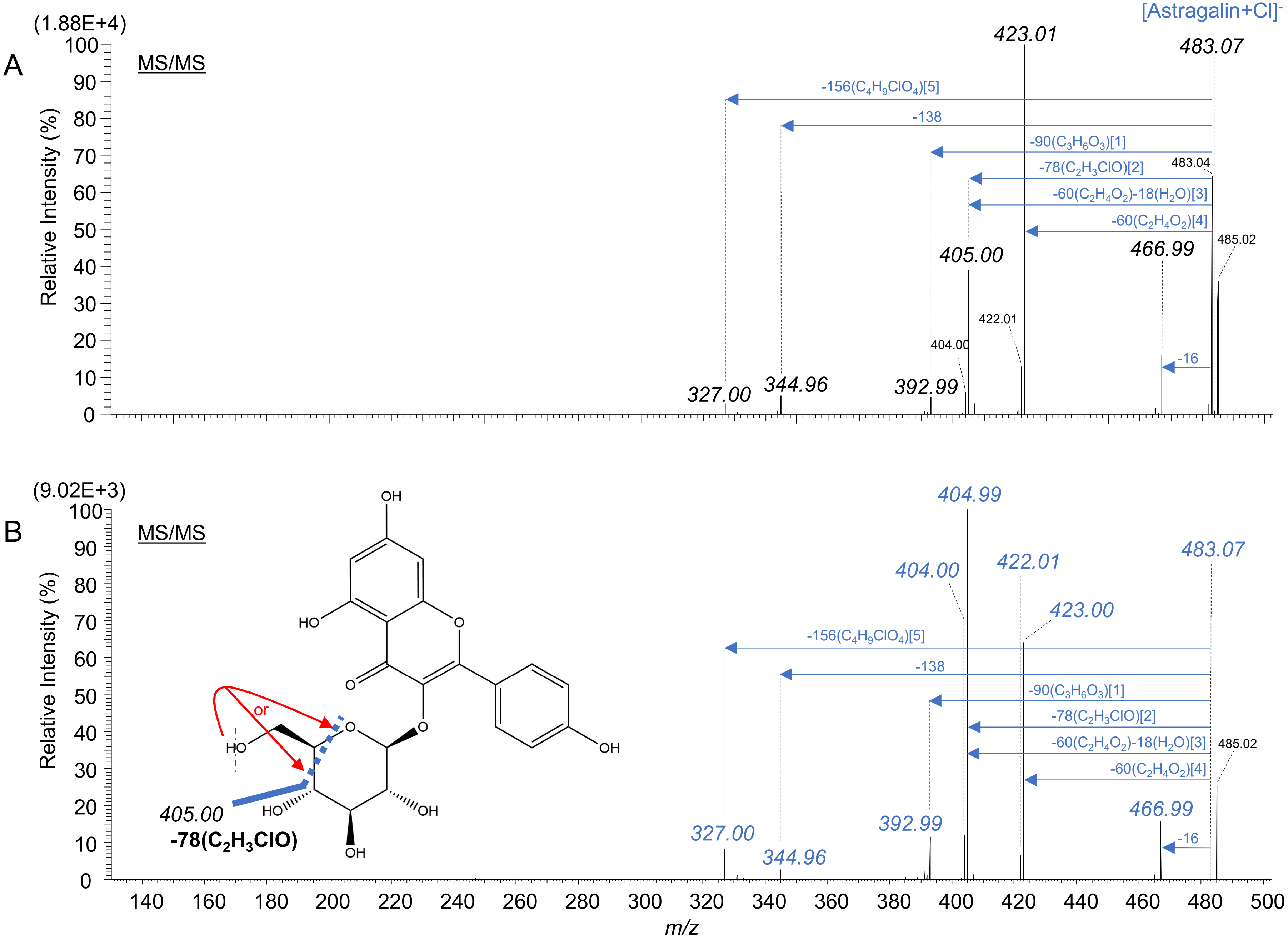

Figure S4.4. (A) PicoPPESI-MS/MS negative ion mode spectrum of a putative Astragalin-related peak ([M+Cl]$^-$, *m/z* 483.07) obtained from the rice stigma tissue extracts (see Materials and Methods). Precursor ion *m/z* 483.0701; selector gate range *m/z* 482.62-483.52. Fragmentation was induced with a 28 % normalized collision energy. (B) PicoPPESI-MS/MS negative ion mode spectrum of a putative Astragalin-related peak ([M+Cl]$^-$, *m/z* 483.07) obtained from 0.5mM Astragalin (Kaempferol 3-β-D-glucopyranoside) solution containing 100 mM NaCl. Precursor ion *m/z* 483.0701; selector gate range *m/z* 482.62-483.52. Fragmentation was induced with a 35 % normalized collision energy. The eight signals with *m/z* shown in blue in B matched with A. According to the MS/MS spectra, the precursor ion at *m/z* 615.03 was identified as [Astragalin+Cl]$^-$.

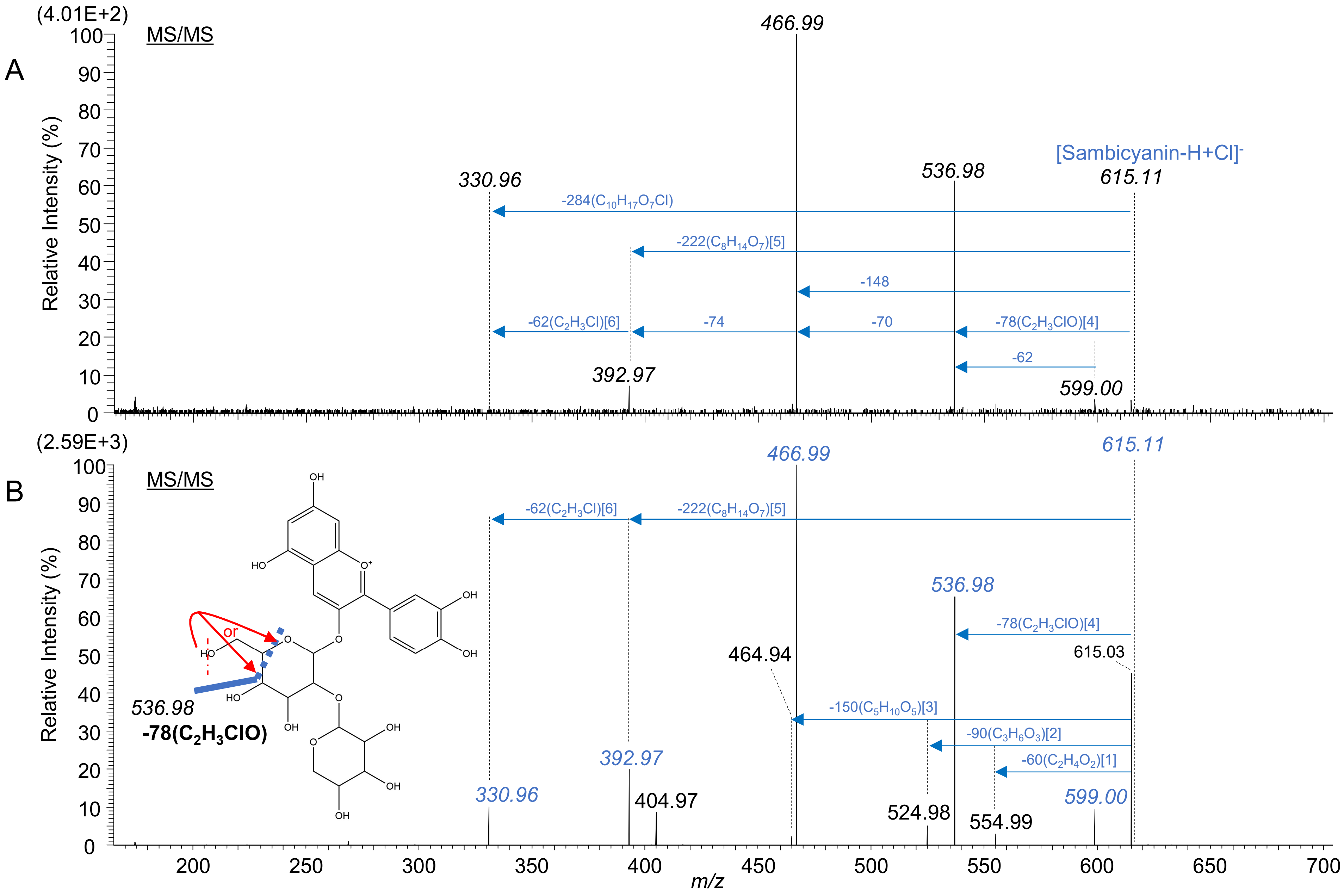

Figure S4.5. (A) PicoPPESI-MS/MS negative ion mode spectrum of a putative Sambicyanin-related peak ($[M-H+Cl]^-$, *m/z* 615.11) obtained from the rice stigma tissue extracts (see Materials and Methods). Precursor ion *m/z* 615.1115; selector gate range *m/z* 614.66-615.56. Fragmentation was induced with a 30 % normalized collision energy. (B) PicoPPESI-MS/MS negative ion mode spectrum of a putative Sambicyanin-related peak ($[M-H+Cl]^-$, *m/z* 615.11) obtained from the 1:1 mixture of 0.71 mM Sambicyanin (Cyanidin 3-sambubioside) chloride in MeOH and 200 mM NaCl solution. Precursor ion *m/z* 615.1115; selector gate range *m/z* 614.66-615.56. Fragmentation was induced with a 30 % normalized collision energy. The five signals with *m/z* shown in blue in B matched with A. According to the MS/MS spectra, the precursor ion at *m/z* 615.03 was identified as $[Sambicyanin-H+Cl]^-$.

[Astragalin+Cl]-

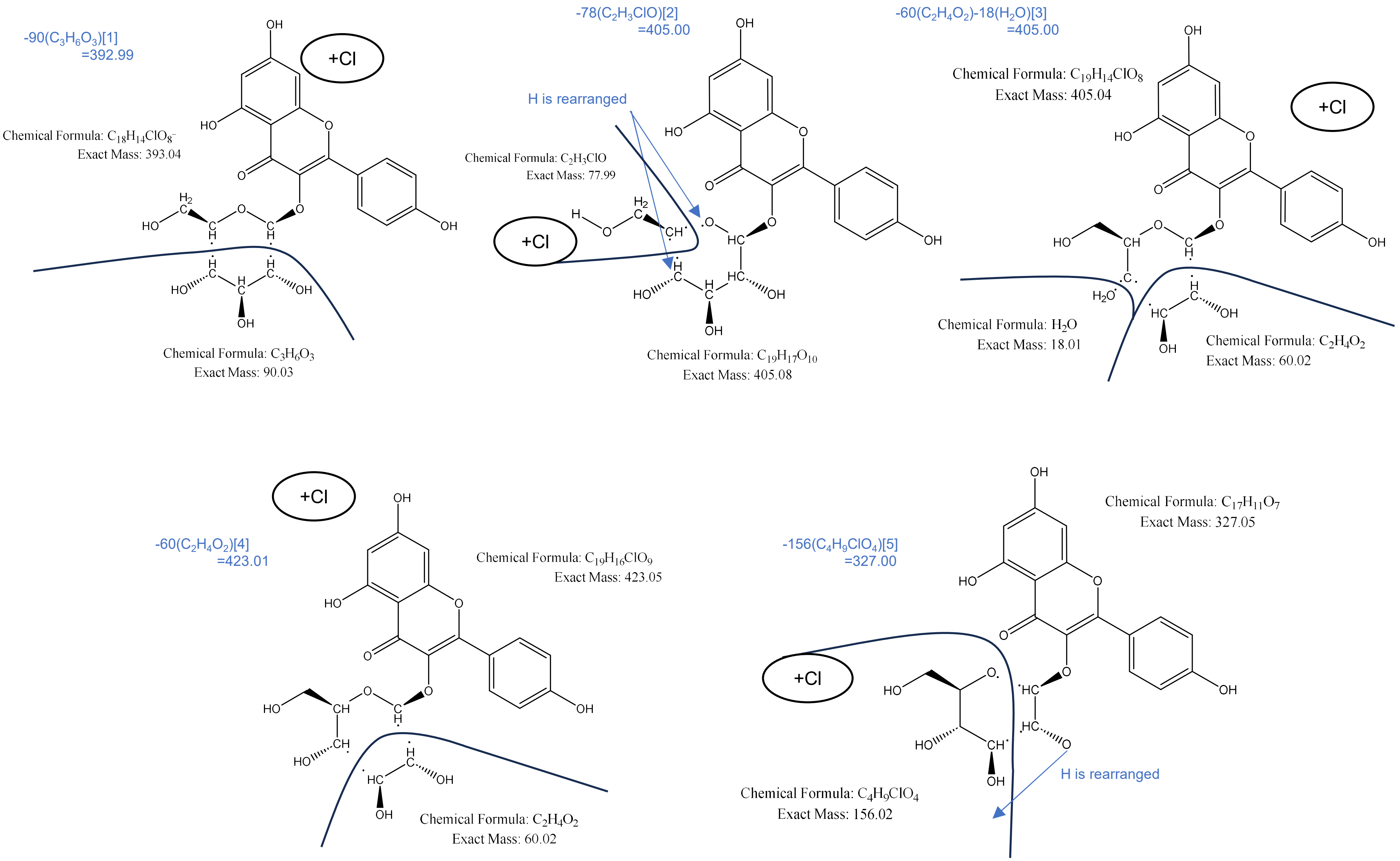

Figure S4.6. Putative cleavage patterns of Astragalin-related ions ([M+Cl]-) during MS/MS analysis. Rearrangement possibilities of H from–HO group is indicated in blue. These patterns indicate the rationalization of the origin of the fragments shown in Fig. S4.4 observed experimentally.

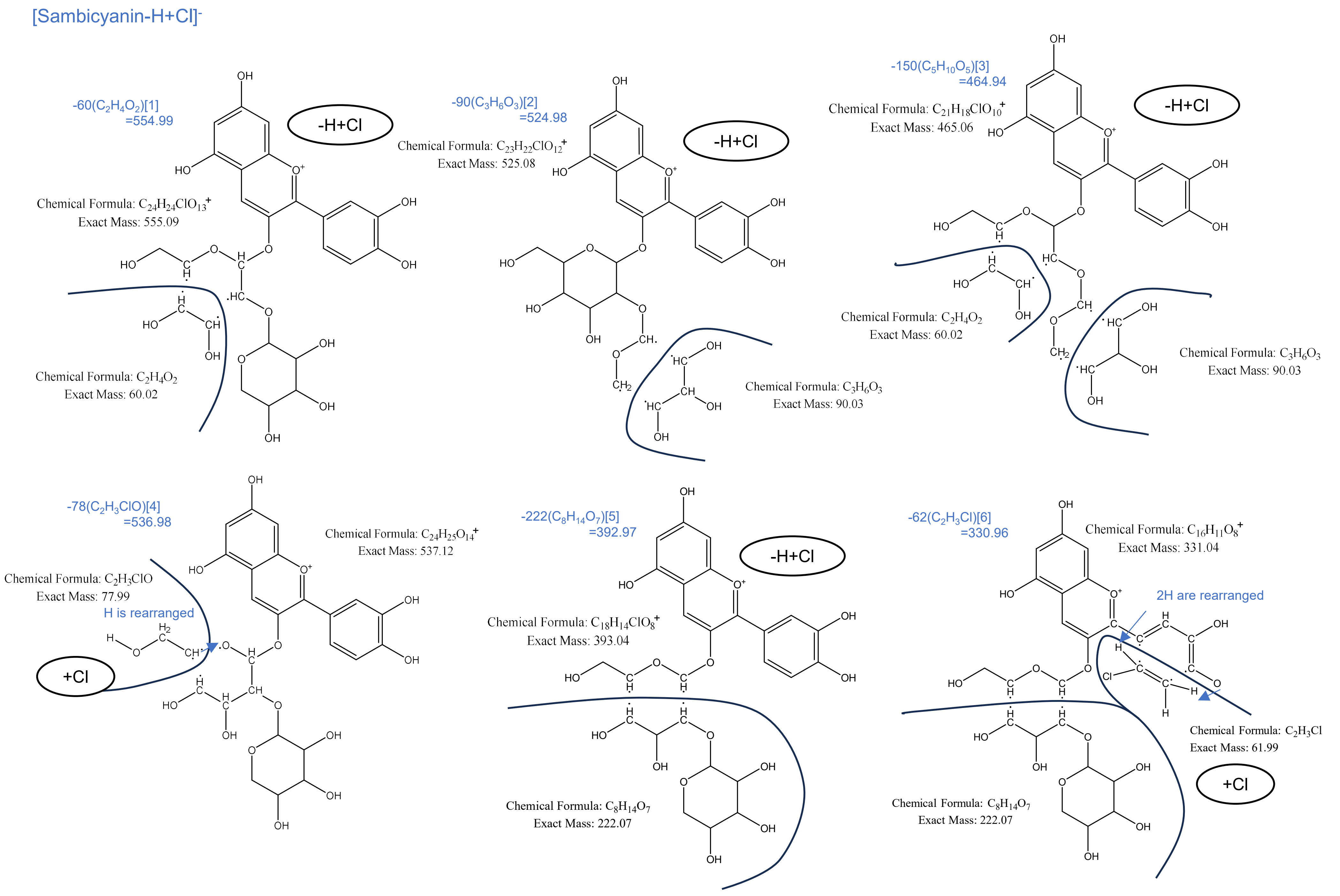

Figure S4.7. Putative cleavage patterns of Sambicyanin-related ions ([M-H+Cl]-) during MS/MS analysis. Rearrangement possibilities of H from–HO group is indicated in blue. These patterns indicate the rationalization of the origin of the fragments shown in Fig. S4.5 observed experimentally.

[Astragalin+Cl]- [2]
[Sambicyanin-H+Cl]- [4]

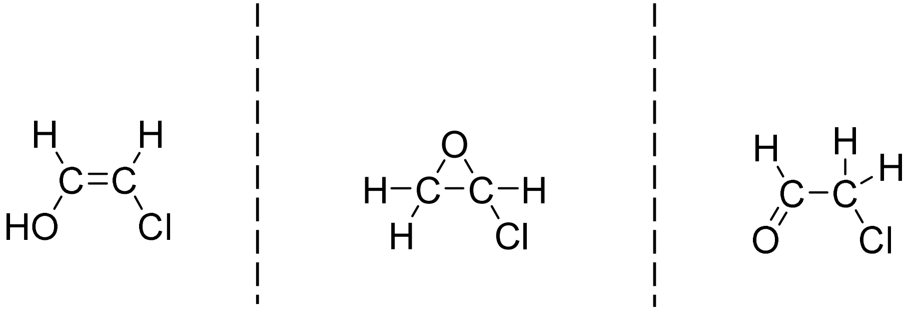

Chemical Formula: C_2H_3ClO
Exact Mass: 77.99

[Astragalin+Cl]- [3][4]
[Sambicyanin-H+Cl]- [1]

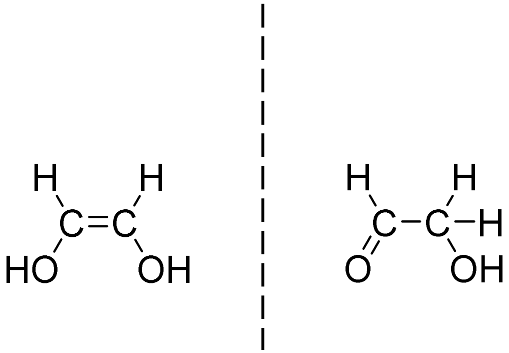

Chemical Formula: $C_2H_4O_2$
Exact Mass: 60.02

[Astragalin+Cl]- [1]
[Sambicyanin-H+Cl]- [2]

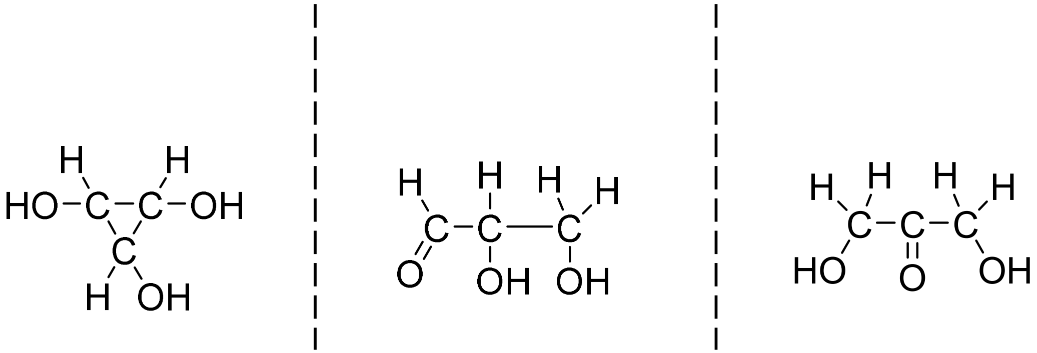

Chemical Formula: $C_3H_6O_3$
Exact Mass: 90.03

Figure S4.8. Putative molecular structures of neutral fragments generated by picoPPESI-MS/MS analysis.

Supplementary References

1. Heslop-Harrison, J. An interpretation of the hydrodynamics of pollen. *Am J Bot* **66**, 737-743 (1979). https://doi.org:10.2307/2442418
2. Slatyer, R. O. *Plant-water relationships*. (Academic Press, 1967).
3. Passioura, J. B. The Meaning of Matric Potential. *J Exp Bot* **31**, 1161-1169 (1980). https://doi.org:10.1093/jxb/31.4.1161
4. Nonami, H. & Boyer, J. S. Origin of growth-induced water potential: Solute concentration is low in apoplast of enlarging tissues. *Plant Physiology* **83**, 596-601 (1987). https://doi.org:10.1104/pp.83.3.596
5. Wada, H. *et al.* Direct evidence for dynamics of cell heterogeneity in watercored apples: turgor-associated metabolic modifications and within-fruit water potential gradient unveiled by single-cell analyses. *Hort. Res.* **8**, 187 (2021). https://doi.org:10.1038/s41438-021-00603-1
6. Watanabe, K. Studies on the germination of grass pollen I liquid exudation of the pollen on the stigma before germination. *Bot. Mag. Tokyo.* **68**, 40-44 (1955). https://doi.org:10.15281/jplantres1887.68.40
7. Wada, H., Shackel, K. A. & Matthews, M. A. Fruit ripening in *Vitis vinifera*: apoplastic solute accumulation accounts for pre-veraison turgor loss in berries. *Planta* **227**, 1351-1361 (2008). https://doi.org:10.1007/s00425-008-0707-3
