# Supplementary material for: ‘Roly-poly toy’ motion during pollen exudation promotes rapid pollen adhesion in rice
Source: Commun Biol. 2025 Apr 18;8:608. doi: 10.1038/s42003-025-08018-7 (PMC12008421; doi:10.1038/s42003-025-08018-7)
Supplement: Supplementary file 2 — Description of Additional Supplementary Files [file 42003_2025_8018_MOESM2_ESM.pdf]

Description of Additional Supplementary Files

File name: Supplementary Data 1

Description: The source data behind the graphs and data statement in the Results in the paper.

File name: Supplementary Data 2

Description: List of metabolites detected in pollen exudates, mature pollen grains, and stigma cells in rice using picoPPESI-MS in negative ion mode.

File name: Supplementary Movie 1
Description: The rapid pollination process with pollen exudation followed by roly-poly toy motion in rice pollen grains. In the file, each event, pollen capture, the initiation of pollen exudation, and roly-poly toy like motion can be seen at 00'10", 00'23", and 00'24-26", respectively.

File name: Supplementary Movie 2
Description: Video recorded for'Roly-poly toy' motions in two pollen grains. The file was videotaped from the top of the pollen grains. Also see Supplementary Figure S3.

File name: Supplementary Movie 3
Description: Artificial pollination followed by nanolitre exudate collection using a cell pressure probe. After the exudate collection, on-site picolitre fluid metabolomics using picolitre pressure-probe electrospray-ionization mass spectrometry (picoPPESI-MS) was carried out.
