# Supplementary material for: ‘Roly-poly toy’ motion during pollen exudation promotes rapid pollen adhesion in rice
Source: Commun Biol. 2025 Apr 18;8:608. doi: 10.1038/s42003-025-08018-7 (PMC12008421; doi:10.1038/s42003-025-08018-7)
Supplement: Supplementary file 8 — Reporting summary [file 42003_2025_8018_MOESM8_ESM.pdf]

nature portfolio

Corresponding author(s): Hiroshi Wada

Last updated by author(s): Mar 17, 2025

Reporting Summary

Nature Portfolio wishes to improve the reproducibility of the work that we publish. This form provides structure for consistency and transparency in reporting. For further information on Nature Portfolio policies, see our Editorial Policies and the Editorial Policy Checklist.

Statistics

For all statistical analyses, confirm that the following items are present in the figure legend, table legend, main text, or Methods section.

n/a	Confirmed	
☐	☒	The exact sample size (n) for each experimental group/condition, given as a discrete number and unit of measurement
☐	☒	A statement on whether measurements were taken from distinct samples or whether the same sample was measured repeatedly
☐	☒	The statistical test(s) used AND whether they are one- or two-sided<br>*Only common tests should be described solely by name; describe more complex techniques in the Methods section.*
☒	☐	A description of all covariates tested
☒	☐	A description of any assumptions or corrections, such as tests of normality and adjustment for multiple comparisons
☐	☒	A full description of the statistical parameters including central tendency (e.g. means) or other basic estimates (e.g. regression coefficient) AND variation (e.g. standard deviation) or associated estimates of uncertainty (e.g. confidence intervals)
☐	☒	For null hypothesis testing, the test statistic (e.g. F, t, r) with confidence intervals, effect sizes, degrees of freedom and P value noted<br>*Give P values as exact values whenever suitable.*
☒	☐	For Bayesian analysis, information on the choice of priors and Markov chain Monte Carlo settings
☒	☐	For hierarchical and complex designs, identification of the appropriate level for tests and full reporting of outcomes
☒	☐	Estimates of effect sizes (e.g. Cohen's d, Pearson's r), indicating how they were calculated

Our web collection on statistics for biologists contains articles on many of the points above.

Software and code

Policy information about availability of computer code

Data collection	Digital microscope (KH-8700, HIROX Co. Ltd., Tokyo, Japan) for microscopic observations.<br>Cell pressure probe with a pressure transducer (XTM-190SM-500SG, Kulite, USA) for cell turgor measurement.<br>On-site cell metabolomics, picolitre pressure-probe electrospray-ionization mass spectrometry (picoPPESI-MS) with Orbitrap mass spectrometers (Q-Exactive and Orbitrap Elite, ThermoFisher Scientific Inc., MA, USA), picoPPESI-MS/MS, picoPPESI-MS/MS/MS).
Data analysis	Raw data analysis and the generation of a heatmap: Microsoft Excel 2019.<br>Data statistics: JMP (version 12.1.0; SAS Institute Inc., Cary, NC, USA).<br>Video cutout and insertion of video text in the movies collected during the microscopic observations, capturing images in the original files, and generation of video data as supplementary information: Adobe Photoshop and Premiere Elements 2021.<br>Image analyses for pollen diameter and contact angle formed in the foot-like structure in the captured images: ImageJ software (https://imagej.nih.gov/ij/).<br>Pollen motion analysis by identifying the pixel coordinates corresponding to the center of pollen grains: ImageJ software.<br>Generation of a graph on time-course of changes in pollen diameter after pollen capture: SigmaPlot for Windows Version 14.5.<br>Generation of the cartoon files: Microsoft PowerPoint 2019.<br>PicoPPESI-MS analysis and chemical identification: Thermo Xcalibur software (ThermoFisher Scientific) and METLIN online metabolomics database (https://metlin.scripps.edu/index.php) and the Thing Metabolome Repository (http://metabolites.in/things/).

For manuscripts utilizing custom algorithms or software that are central to the research but not yet described in published literature, software must be made available to editors and reviewers. We strongly encourage code deposition in a community repository (e.g. GitHub). See the Nature Portfolio guidelines for submitting code & software for further information.

Data

Policy information about availability of data

All manuscripts must include a data availability statement. This statement should provide the following information, where applicable:

- Accession codes, unique identifiers, or web links for publicly available datasets
- A description of any restrictions on data availability
- For clinical datasets or third party data, please ensure that the statement adheres to our policy

All the data generated in this study are available in the paper and Supplementary Information (Supplementary Discussion 1, Supplementary Tables S1, Supplementary Figures S1-S4, and Supplementary Movies 1, 2, and 3). The movies of 'roly-poly toy'-like rice pollen grain motion on exudates are provided in Supplementary Movies 1 and 2. All other data are available from the corresponding author on reasonable request.

Research involving human participants, their data, or biological material

Policy information about studies with human participants or human data. See also policy information about sex, gender (identity/presentation), and sexual orientation and race, ethnicity and racism.

Reporting on sex and gender	n/a
Reporting on race, ethnicity, or other socially relevant groupings	n/a
Population characteristics	n/a
Recruitment	n/a
Ethics oversight	n/a

Note that full information on the approval of the study protocol must also be provided in the manuscript.

Field-specific reporting

Please select the one below that is the best fit for your research. If you are not sure, read the appropriate sections before making your selection.

☒ Life sciences ☐ Behavioural & social sciences ☐ Ecological, evolutionary & environmental sciences

For a reference copy of the document with all sections, see nature.com/documents/nr-reporting-summary-flat.pdf

Life sciences study design

All studies must disclose on these points even when the disclosure is negative.

Sample size	Sample size was determined based on the previous studies and experiments in rice pollination. Sample size was indicated in the corresponding figure legend.
Data exclusions	For microscopic observations, pollen grain samples in-focus taken in the video files were only used for the further analyses (pollen diameters and event-based duration measurements). Other pollen grain samples out-of-focus were excluded for the analyses.<br>During the direct turgor determination in intact cells (mature pollen grains and stigmatic papillae) and picolitre fluids (exudates) extraction, samples impaled with microcapillary tips that had lacked hydraulic connection (i.e., tip plugging) were excluded for turgor measurements and picoPPESI-MS analysis following standard practice. Except for these, no data were excluded in other data collections.
Replication	We used eight independent in-focus samples in characterising the changes in pollen diameter change after pollen capture. For the determination of initiation time of each event (initiations of exudation and roly-poly toy-like motion) after pollen capture, 26-29 replicates were used. For the determinations of pollen turgor and pollen diameters, 7 and 8 replicates were used, respectively. For the measurements of contact angle at pollen adhesion and the angle rotated during the rocking motion, 12 and 22 replicates were used, respectively. For picoPPESI-MS analysis, 7-11 replicates obtained from 4–5 independent plants were used. For chemical identifications for unknown signals in stigma samples, three biological replications in field-grown rice plants were used to collect the tissue extracts.
Randomization	All samples were randomly collected.
Blinding	Blinding was not applied in this study. Experimental results are objectively collected and not subjective.

Reporting for specific materials, systems and methods

We require information from authors about some types of materials, experimental systems and methods used in many studies. Here, indicate whether each material, system or method listed is relevant to your study. If you are not sure if a list item applies to your research, read the appropriate section before selecting a response.

Materials & experimental systems

n/a	Involved in the study
☒	☐ Antibodies
☒	☐ Eukaryotic cell lines
☒	☐ Palaeontology and archaeology
☒	☐ Animals and other organisms
☒	☐ Clinical data
☒	☐ Dual use research of concern
☐	☒ Plants

Methods

n/a	Involved in the study
☒	☐ ChIP-seq
☒	☐ Flow cytometry
☒	☐ MRI-based neuroimaging

Dual use research of concern

Policy information about dual use research of concern

Hazards

Could the accidental, deliberate or reckless misuse of agents or technologies generated in the work, or the application of information presented in the manuscript, pose a threat to:

No	Yes
☒	☐ Public health
☒	☐ National security
☒	☐ Crops and/or livestock
☒	☐ Ecosystems
☒	☐ Any other significant area

Experiments of concern

Does the work involve any of these experiments of concern:

No	Yes
☒	☐ Demonstrate how to render a vaccine ineffective
☒	☐ Confer resistance to therapeutically useful antibiotics or antiviral agents
☒	☐ Enhance the virulence of a pathogen or render a nonpathogen virulent
☒	☐ Increase transmissibility of a pathogen
☒	☐ Alter the host range of a pathogen
☒	☐ Enable evasion of diagnostic/detection modalities
☒	☐ Enable the weaponization of a biological agent or toxin
☒	☐ Any other potentially harmful combination of experiments and agents

Plants

Seed stocks	Rice (cv. Koshihikari) seeds were obtained from NARO Genebank.
Novel plant genotypes	The manuscript is a physiological work on rice pollination with a leading cultivar and has no novel plant genotypes with new plant breeding techniques.
Authentication	In this manuscript, there are no novel plant genotypes.
